# Burden of intimate partner violence, mental health issues, and help-seeking behaviors among women in Nepal

**DOI:** 10.1177/17455057251326416

**Published:** 2025-03-18

**Authors:** Monna Kurvinen, Anna Mia Ekström, Keshab Deuba

**Affiliations:** 1 Department of Global Public Health, Karolinska Institutet, Stockholm, Sweden; 2 Public Health and Environment Research Centre (PERC), Lalitpur, Nepal; 3 Centre for International Health (CIH), Department of Global Public Health and Primary Care, University of Bergen, Bergen, Norway

**Keywords:** intimate partner violence, IPV, gender-based violence, GBV, mental health, women, help-seeking, NDHS, Nepal

## Abstract

**Background:**

Intimate partner violence (IPV) is the most common form of violence, presenting a significant public health concern, especially for women and girls. Help-seeking can reduce future IPV and mitigate adverse health outcomes, including mental health issues.

**Objectives:**

This study is the first national assessment on IPV, mental health consequences, and associated help-seeking behaviors in Nepal.

**Design:**

A cross-sectional descriptive study.

**Methods:**

Using secondary data from the 2022 Nepal Demographic and Health Survey, this study includes 5178 women aged 15–49 and employs multivariate regression analysis to explore the association between IPV and mental health problems, as well as factors influencing help-seeking behavior for both issues.

**Results:**

Among participants, 31.4% reported ever experiencing IPV, and most (29.4% of all women) in the past 12 months. Of those ever experiencing IPV, 72.0% had not sought help for IPV, and 92.2% of those who did, opted for informal support. A total of 27.6% (*n* = 1427) of female interviewees reported anxiety symptoms, 21.5% (*n* = 1110) depressive symptoms, and 7.1% (*n* = 368) suicidal ideation within the past 2 weeks. These rates were higher among women who had experienced IPV in the past 12 months, with 41.1% reporting anxiety, 33.2% depression symptoms, and 14.1% suicidal ideation. Of the 4194 respondents with symptoms of anxiety, depression, or suicidal ideation who were asked about help-seeking for mental health issues, 19.4% (*n* = 812) had sought help, primarily from informal sources (93.4%, *n* = 759). Emotional IPV in the past 12 months increased the odds of anxiety (adjusted odds ratio (aOR) 3.00, 95% confidence interval (CI) 2.08–4.31), depression (aOR 3.09, 95% CI 2.19–4.37), and suicidal ideation (aOR 1.91, 95% CI 1.20–3.04). Sexual IPV increased the odds of anxiety (aOR 2.88, 95% CI 1.67–4.95) and depression (aOR 2.12, 95% CI 1.32–3.41), while controlling behavior heightened the odds of depression (aOR 2.42, 95% CI 2.02–2.89) and suicidal ideation (aOR 2.24, 95% CI 1.25–4.02).

**Conclusion:**

This nationwide study reveals a high prevalence of IPV and related mental health problems among women in Nepal and a low rate of help-seeking behavior, in particular to formal support structures. Stronger health system responses and empowering informal support are essential to improve referrals and raise awareness for violence survivors.

## Introduction

Intimate partner violence (IPV) is a serious and, at times fatal, public health concern with profound consequences on individuals, families, and communities.^1^ Moreover, it represents a violation of human rights and fundamental freedoms.^2^ As per the World Health Organization (WHO), IPV encompasses actions within an intimate relationship that lead to physical, sexual, or psychological harm.^3^ These actions comprise various forms of maltreatment inflicted by an intimate partner, including physical aggression (such as hitting or kicking), sexual coercion (encompassing non-consensual sexual acts), psychological abuse (such as insults and intimidation) and controlling behaviors (such as isolation and resource restriction).^1,3^ The definition of IPV applies to violence by both current and any former spouses or partners.^3^

The WHOs latest estimate from 2018 indicates that 26% of women aged 15 years and older, who have ever been married or partnered, have experienced sexual and/or physical IPV at least once in their lifetime.^4^ Corresponding statistics reveal that 27% of Nepalese women have encountered physical and/or sexual violence from their current or former intimate partner in their lifetime.^4^ In 2021, during the Coronavirus disease (COVID-19) pandemic, a study conducted by Bhatt et al. revealed that nearly 53% of Nepalese women experienced IPV.^5^ Economic violence emerged as the most prevalent type of IPV, affecting 39% of participants, followed by behavioral control (37%), emotional violence (26%), physical violence (21%), and sexual violence (14%).^5^

Recognizing and understanding the associated risks can offer valuable insights for tailoring care, attention, and prevention strategies, particularly for populations at higher risk.^6^ The impact of IPV disproportionately affects women and girls, with the majority of cases involving male perpetrators and female victims.^1^ The issue is pervasive on a global scale, with particularly high prevalence in low-income countries.^1^ Both in Nepal and globally, factors such as a lower level of education, a history of child abuse, and harmful alcohol use have been associated with an increased risk of IPV.^7–10^ At the relationship level, studies conducted in Nepal have highlighted additional risk factors, including fear of the husband, poor marital communication, and frequent quarrels with the husband, all of which increase the risk of IPV.^5,9^ Antisocial personality disorder in perpetrator^7,8,10^ and unplanned pregnancies are also identified as specific risks for IPV against women.^8^ On a broader community and societal level, factors contributing to the perpetration of IPV include poverty and prevailing acceptance of such violence within the community.^7,10^ Moreover, the absence of laws protecting survivors, insufficient penalties for perpetrators, and gender inequity serves as a risk factor,^10^ along with harmful gender norms.^8–10^

IPV is linked to various mental health issues such as depression, anxiety, post-traumatic stress disorder, self-harm,^11–13^ psychological distress,^12^ and sleep disorders.^11^ Additionally, IPV is prominently observed in women grappling with substance use, eating disorders, and various psychosomatic conditions.^14^ The link between depressive symptoms and IPV is particularly strong, especially in cases of systematic IPV (repeated and diverse forms of IPV).^5^ Women who have encountered IPV are 2.7 times more likely to suffer from mental health issues, including depression and suicidal ideation.^1^ A study from Sri Lanka reported even higher odds, with women experiencing domestic violence being over three times more likely to suffer from depression and more than six times more likely to report suicidal ideation.^15^ Previous research implies that the impact of psychological IPV is comparable to that of physical IPV, except in relation to its effect on suicidal tendencies, which is more prevalent among those experiencing physical IPV.^13^ The simultaneous occurrence of sexual IPV alongside physical and/or psychological IPV is associated with increased depressive symptoms, and a higher incidence of suicide attempts, particularly among those experiencing both physical and psychological IPV.^13^

Help-seeking is the act of searching for or requesting help from others through formal or informal means.^16^ It involves a complex decision-making process triggered by a problem that challenges personal abilities.^17^ Engaging in help-seeking has the potential to reduce the risk of future IPV and mitigate adverse health outcomes, including mental health problems.^18^ For women experiencing IPV, social support has a significant protective effect on their quality of life and in reducing depression.^19^

Globally, the availability of support for women facing IPV varies based on the context and includes support services offered by healthcare providers at different levels, multi-agency support, or One-stop crisis centers services.^20^ These measures typically connect survivors with legal, police, housing, and financial services, often incorporating psychological support as well.^20^ In Nepal, women can access a variety of support services, encompassing physical and mental health services, social services, and assistance from the police and legal authorities.^21^ However, the availability and quality of these services differ based on the specific location within Nepal.^21^ Additionally, women may face several challenges, such as limited awareness of available services, fear of the consequences of disclosing abuse, lack of material resources, and personal barriers.^22^ In 2011, hospitals implemented One-stop crisis management centers to offer comprehensive services to survivors of gender-based violence.^23^ These services include immediate medical treatment, counseling for psychosocial well-being, legal guidance, safe shelter, security, and rehabilitation support, as well as education and empowerment initiatives.^23^ In 2023, there were 88 One-stop crisis management centers established across the country.^23^ Several obstacles to seeking help for IPV have been identified, encompassing issues such as limited awareness of service options, access challenges, potential consequences of disclosure, insufficient material resources, personal barriers, and systemic failures.^22^

Mental health care, specifically, is delivered through psychiatry units in medical colleges, provincial government hospitals, and a few private healthcare facilities.^24^ In total, there are 25 psychiatric facilities in Nepal designed for in-patient care, collectively offering 500 beds.^24^ Traditional healing practices are also prominent, and many initially seeking help from traditional, religious, or faith healers for mental health concerns.^24–26^

Despite significant efforts over the past decade to combat violence against women in Nepal through policy and programmatic interventions, substantial research gaps remain concerning IPV, particularly its impact on mental health within the country. The 2022 Nepal Demographic and Health Survey (NDHS), which is analyzed in this study, represents the first national, population-based survey conducted by Nepal on IPV and its mental health repercussions. This survey’s scope allows for findings to be generalized at national and subnational (provincial) level. Previous studies in Nepal have been constrained by either limited sample sizes or a focus on specific geographic regions. Additionally, this survey is notable for incorporating mental health issues within the NDHS for the first time. Unlike earlier NDHS surveys that collected data solely on IPV experienced by ever-married women, this survey round expanded its scope to include never-married women who have reported IPV from current or former intimate partners. This study not only addresses the persistent research gap concerning IPV and its impact on women’s mental health but also investigates whether Nepalese women experiencing IPV seek help or support, and if so, what types of support they access. It further explores the factors influencing their help-seeking behaviors, providing insights that could enhance existing programs or interventions aimed at addressing the dual challenge of IPV and mental health issues. This investigation aligns with United Nations Sustainable Development Goals 3 and 5, assessing the current state and potential programmatic implications for the elimination of violence against women, including IPV.

## Methodology

### Study design and setting

This quantitative study utilizes a cross-sectional descriptive approach to explore the association between IPV and women’s mental health in Nepal. The reporting follows the STROBE guidelines for cross-sectional studies (see Supplemental Material for the completed STROBE checklist).^27^ The analysis is based on secondary data from the 2022 NDHS. The NDHS is a population-based survey implemented as part of the worldwide DHS program.^28^ This program collaborates with governments to collect and disseminate essential information about people, their health, and health systems, ensuring quality and standardizations at both regional and global levels.^29^ Study setting consists of households in both urban and rural areas across Nepal’s seven provinces.^28^ Nepal is a lower-middle-income country, with around 65% of its population residing in urban areas^28^ and home to 142 distinct castes and ethnic groups.^30^ Moreover, the country’s almost decade-long civil war, from February 1996 to November 2006, resulted in the deaths of more than 17,000 people and displaced thousands of civilians from rural and remote areas to urban centers in Nepal.^31^ During this period, the already fragile healthcare system was further weakened by the war.

### Study tool

The survey employed four distinct questionnaires: the Household Questionnaire, the Woman’s Questionnaire, the Man’s Questionnaire, and the Biomarker Questionnaire, all of which can be found in the final report of the 2022 NDHS.^28^ Specifically, the Woman’s Questionnaire was run to gather information from women aged 15–49, covering a range of health-related topics.^28^ For this study, emphasis was placed on exploring the modules on background characteristics, domestic violence, and mental health.

In the domestic violence module, data were gathered from women aged 15–49 concerning their encounters with violence, including violence committed by current and former husbands or other intimate partners.^28^ To specifically address IPV, women who had ever been married were interviewed on incidents involving their current and former husbands.^28^ Similarly, never-married women were asked about instances of violence involving their current and former intimate partner.^28^ The mental health module utilized the Patient Health Questionnaire (PHQ-9), which has been validated in the adult population of Nepal for screening depression symptoms.^28^ Additionally, it included the Generalized Anxiety Disorder 7 scale (GAD-7) for screening anxiety symptoms, both of which incorporate inquiries about seeking care and treatment.^28^

Once all questionnaires were finalized in English, they underwent translation into Nepali, Maithili, and Bhojpuri.^28^ The pretesting of the questionnaires occurred between October 6 and October 10, 2021.^28^ Subsequent modifications to the questionnaires were made based on insights gained from the pretesting exercise.^28^

### Sampling strategy, study population, and sample size

The survey employed a two-stage cluster sampling approach, which was stratified by urban and rural areas.^28^ First, a total of 476 clusters were selected through probability-proportional-to-size selection with independent selection in each stratum.^28^ These clusters were drawn from the 14 strata, which were formed by dividing each of the 7 provinces into urban and rural areas.^28^ Among the clusters, 248 were from urban and 228 from rural areas.^28^ The sampling frame used is a revised version of the frame derived from the 2011 Nepal Population and Housing Census.^28^ The census frame encompasses a comprehensive list of Nepal’s 36,030 sub-wards, each categorized by residence type (urban or rural), with the measure of size being the number of households.^28,32^

In all selected clusters, a household listing was conducted.^28^ The resulting list of households served as the sampling frame for the second-stage selection of households.^28^ The survey utilized a systematic sampling approach to choose 30 households from each cluster, resulting in a total sample size of 14,280 households.^28^ This included 7440 from urban and 6840 from rural areas.^28^ Within this sample, every second household was selected for the domestic violence and mental health modules, resulting in a total of 7140 selected households.^28^

All women aged 15–49 who were permanent residents of the selected households or visitors staying in the households the night before the survey were eligible for interviews.^28^ The domestic violence module was administered to a subsample of households selected for the entire NDHS 2022. Sample weights were applied to ensure that this subsample, which included only one woman from each household, was nationally representative, with further details available in the 2022 NDHS final report.^28^

Only one eligible woman per household was randomly selected for the domestic violence module, and it was only conducted if privacy could be ensured.^28^ Out of the 14,845 eligible women for the module, 5178 were interviewed, constituting the final sample used for the analysis.^28^ All women interviewed for the domestic violence module were also asked about their background characteristics and received the mental health module.^28^

### Data collection

The survey was conducted between January 5, 2022 and June 22, 2022.^28^ Computer-assisted face-to-face interviews served as the data collection tool. The data collection process for the 2022 NDHS involved 19 teams, each comprising a supervisor, 1 male interviewer, 3 female interviewers, and 1 biomarker specialist. Prior to field work, the staff underwent 2 weeks of training, followed by 4 days of field practice.

### Variables

#### Independent variables

Participants provided information regarding their age, type of place of residence (urban/rural), province of residence, educational level, employment status, race/ethnicity, marital status, household wealth, and the alcohol consumption patterns of both partners and women. Means and medians were utilized to describe the variation in age, whereas frequencies and percentages were employed to illustrate the variation in other sociodemographic and behavioral characteristics.

To assess household wealth, households received scores based on their ownership of consumer goods and housing characteristics, such as televisions, water source, and toilet facilities.^28^ Then, each household member was assigned the household’s score and was ranked accordingly.^28^ Finally, the population was divided into five equal groups, each representing 20% of the total, to create national wealth quintiles.^28^ The first two wealth quintiles were combined into the “poor” category, the third and fourth wealth quintiles are grouped together as “middle,” and the fifth wealth quintile is classified as “rich.” Partner drinking was defined as whether the partner drank alcohol (yes/no) and the frequency of drunkenness in the past month (never/sometimes/often). Women’s alcohol consumption in the past month was described in four categories: no alcohol consumption, consuming alcohol 1–10 days, 11–24 days versus 25 days to daily in the past month.

The independent variables of concern pertain to various forms of violence experienced by women, perpetrated by their intimate partners—emotional, physical, and sexual violence, and/or controlling behavior. To assess IPV, eligible (ever married or having had an intimate partner) women aged 15–49 who were randomly selected and interviewed were asked about their lifetime experiences of IPV. Women who reported ever experiencing IPV were further queried about facing IPV in the past 12 months. The items used to measure various forms of IPV are presented in Supplemental Appendix Table A1.

Each type of IPV was dichotomously coded, and separate variables were created for experiencing IPV ever and in the last 12 months. For instance, if at least one of the seven items for physical violence was reported, it was coded 1 for experiencing physical violence. Similarly, if at least one of the seven items for physical violence were reported happening in the last 12 months, it was coded as 1 for experiencing physical IPV in the last 12 months. Additionally, binary variables of experiencing any type of IPV ever and in the last 12 months were created. If at least one of the four types of IPV was reported, it was coded 1 for any type of IPV.

#### Primary outcome of interest

The primary outcomes of interest in this study encompass mental health problems, specifically including anxiety, depression, and suicidal ideation. To evaluate anxiety symptoms, the mental health module employed the GAD-7, a set of seven items designed to measure the primary aspect of anxiety: persistent and impairing worry.^33,34^ Additionally, the GAD-7 encompasses features related to three other prevalent anxiety disorders: panic disorder, social anxiety disorder, and posttraumatic stress disorder.^33,34^ To evaluate depression symptoms, the module included nine items from the PHQ-9, which align with the diagnostic criteria for depression as outlined in the *Diagnostic and Statistical Manual of Mental Disorders*.^34,35^ Additionally, the mental health module featured a question from the PHQ-9 that specifically inquired about suicidal ideation.^34^

Responses to questions about anxiety and depression were rated on a 4-point Likert scale, reflecting the frequency of experiencing symptoms in the past 2 weeks (never, rarely, often, or always). The severity levels of anxiety were categorized based on the following cut-offs: a score of 0–4 represented minimal anxiety, 5–9 indicated mild anxiety, 10–14 signified moderate anxiety, and a score of 15 or greater was classified as severe anxiety. Similarly, total scores of 5, 10, 15, and 20 served as cut-offs indicating mild, moderate, moderately severe, and severe depression. These chosen thresholds align with widely accepted standards.^33,35^

To investigate further the variations in mental health symptoms based on the type of IPV and sociodemographic characteristics, the outcome variables were recoded into a binary variable distinguishing between individuals with no reported anxiety or depressive symptoms (coded as 0) and those reporting anxiety and/or depressive symptoms (coded as 1). Specifically, individuals with a score of four or higher on the GAD-7 were assigned a code of 1 to represent anxiety, while those scoring 5 or higher on the PHQ-9 were assigned a code of 1 to indicate depression. Additionally, suicidal ideation was categorized as a binary variable, indicating whether a person had experienced suicidal thoughts during the past 2 weeks (coded as 1) or not (coded as 0).

#### Secondary outcome of interest

Women who reported experiencing physical and/or sexual IPV were asked a dichotomous question regarding whether they had ever sought help to stop the violence. Dichotomous variables were created, with individuals not seeking any help assigned a code of 0, while those seeking help from any resource were assigned a code of 1. Additionally, for those who indicated seeking help to stop the violence, three variables were generated to explore differences in women’s help-seeking patterns based on different forms of IPV: *formal help-seeking, informal help-seeking, and both formal and informal help-seeking*. Individuals seeking help from only professionals including doctors, police, lawyers, or social work organizations were coded as seeking formal help. Those who sought help only from their natal family, their husband’s or partner’s family, friends, neighbors, religious leaders, or others were coded as seeking informal help. Furthermore, individuals who sought help from informal and formal sources were categorized as seeking help from both.

Similarly, women seeking help specifically for mental health issues were categorized as dichotomous variables: those actively seeking help and those not seeking help. Those seeking help were further categorized into three groups: *formal help-seeking, informal help-seeking, and both formal and informal help-seeking*. Women seeking help from medical personnel, social service organizations, social workers, or community health workers were categorized as seeking formal help. Those seeking help from current or former partners, family members, friends, neighbors, religious leaders, or others were categorized as seeking informal help. Women seeking help from both were categorized as seeking both informal and formal help.

### Statistical analysis

Within the dataset, values labeled as “not applicable” signify questions not intended to be asked based on the questionnaire’s flow. On the other hand, “missing” designates variables that should have a response, but due to interviewer error, the question was not asked. Both “not applicable” and “missing” values were excluded from the analysis. Data analysis was conducted using the statistical software STATA 18 (Stata Corp LLC, College Station, TX, Texas).

Initially, descriptive statistics were employed to summarize the key characteristics of the study population, involving the calculation of frequencies, percentages, means, and standard deviations. Descriptive statistics were also used to describe the prevalence of IPV, mental health issues (anxiety, depression, and suicidal ideation), and help-seeking behavior for IPV and mental health issues. This included univariate analysis. Second, bivariate logistic regression was conducted to identify potential confounders linked with the outcome. Only sociodemographic and behavioral characteristics with a *p*-value of 0.2 or lower in bivariate logistic regression were considered for inclusion in the multivariate logistic regression models assessing associations between independent and outcome variables. The *p*-value cutoff of <0.20 was chosen based on common practice.^36,37^

Third, multivariate regression analysis was employed to identify the association between IPV and mental health problems, as well as to explore the factors associated with seeking help for both IPV and mental health issues. Predictors were assessed in a single model for each outcome. Additionally, separate models were created to analyze the association between ever experiencing IPV and experiencing IPV in the past 12 months in relation to mental health problems. The accuracy and precision of the multivariate logistic regression models were assessed using the number of events per variable (EPV). EPV values of 10 or greater were considered acceptable, reducing the likelihood of obtaining misleading findings.^38,39^

To ensure national representativeness despite the nonproportional allocation of the sample, sample weights were applied. These weights adjusted for both disproportionate sampling and non-response.^28^ All variables were analyzed using a 95% confidence interval (CI), and a significance level of 0.05 was applied to determine the final model’s statistical significance.

### Ethical considerations

Following the WHOs ethical guidelines for gathering information on violence against women, only one eligible woman per household was chosen randomly for the module.^28,32^ The module was excluded if privacy could not be ensured.^29^ For both ethical and safety considerations, it was crucial to not present the survey to the household as a study focused on violence.^32^ In this study, questions regarding IPV were integrated into the Woman’s Questionnaire, which primarily aimed to gather information on basic demographic and health indicators. However, the women themselves were fully informed about the nature of the questions.^28,32^ Interviewees were permitted to pause the interview at any point or skip any questions they preferred not to answer.^34^ Answers were treated with strict confidentiality.^34^

The research team members responsible for data collection were carefully selected and underwent specialized training, accompanied by ongoing support.^28^ Respondents presenting symptoms of suicidal ideation or scoring 10 or higher on the PHQ scale were offered mental health service referrals for further consultation.^34^

## Results

### Sociodemographic and behavioral characteristics

A total of 5178 women aged 15–49 were included in this study. Table 1 provides a summary of participants’ sociodemographic characteristics, taking sample weights into account. Unweighted numbers are presented in Supplemental Appendix Table A2. The mean ± standard deviation age of the participants was 29.7 ± 9.7 years. Out of the 5178 women surveyed, 1364 (26.3%) lacked formal education, while 1580 (30.5%) had primary education, 2055 (39.7%) had secondary education, and only 179 (3.5%) had education beyond the secondary level. Additionally, 59.7% (*n* = 3089) of the participants were currently employed. Most women, totaling 68.2% (*n* = 3531), resided in urban areas, and 74.4% (*n* = 3854) were either married or living with a partner.

**Table 1.** Sociodemographic and behavioral characteristics of women aged 15–49 (*n* = 5178).

Variable	Mean (range)	SD
Age	29.7 (15–49)	9.7
	*N*	%
Educational level		
No education	1364	26.3
Primary	1580	30.5
Secondary	2055	39.7
Higher than secondary	179	3.5
Employment status		
Currently employed	3089	59.7
Worked in the past 12 months (but not currently)	655	12.6
Not employed in the past 12 months	1434	27.7
Type of place of residence		
Urban	3531	68.2
Province of residence		
Koshi	877	17.0
Madhesh	1042	20.1
Bagmati	1037	20.0
Gandaki	496	9.6
Lumbini	947	18.3
Province of residence		
Karnali	324	6.2
Sudurpashchim	455	8.8
Type of place of residence		
Rural	1647	31.8
Ethnicity		
Brahmin/Chhetri (Hill/Terai)	1493	28.9
Other terai caste	750	14.5
Dalit (Hill/Terai)	783	15.1
Janajati/Newar (Hill/Terai)	1911	36.9
Muslim	235	4.5
Other	6	0.1
Marital status		
Never in union	1147	22.1
Married or living with partner	3854	74.4
Widowed	103	2.0
Divorced or separated	75	1.5
Household wealth^a^		
Poor	1915	37.0
Middle	2221	42.9
Rich	1041	20.1
Partner drunk alcohol in the last month		
No	2069	48.7
Yes	2175	51.3
Frequency of partner being drunk in the last month		
Never	675	31.0
Sometimes	1199	55.1
Often	301	13.9
Women consumed alcohol in the last month		
Not consumed	4614	89.1
1–10 days in the past month	440	8.5
11–24 days in the past month	43	0.8
Women consumed alcohol in the last month		
25 days to daily in the past month	81	1.6

All means, standard deviations, counts, and percentages were calculated using sample weights. SD: standard deviation.

a Population is divided into five equal parts based on wealth, each comprising approximately 20% of the population. In this table, the first two wealth quintiles are combined into the “poor” category, the third and fourth wealth quintile are grouped together as “middle,” and the fifth wealth quintile is classified as “rich.”

Geographically, the Madhesh province had the highest number of participants (*n* = 1042), comprising 20.1%, while Karnali had the lowest proportion of participants (*n* = 324, 6.2%). The largest ethnic group among participants was the Brahmin/Chhetri (hill/terai), accounting for 1493 participants (28.9%), while the smallest ethnic group was Muslim with 235 participants (4.5%), and others with only 6 participants (0.1%). Out of all women surveyed, 1915 (37.0%) belonged to poor households, 2221 (42.9%) to middle-income, and 1041 (20.1%) to rich households. Regarding women’s alcohol consumption, a majority (*n* = 4614, 89.1%) had not consumed any alcohol in the past month. Just over half of the interviewed women had a partner who drank alcohol, most (55.1%) reported occasional drunkenness in the past month, while 301 (13.9%) reported frequent drunkenness.

### Experience of IPV, mental health problems, and help-seeking behavior

**Figure 1.**
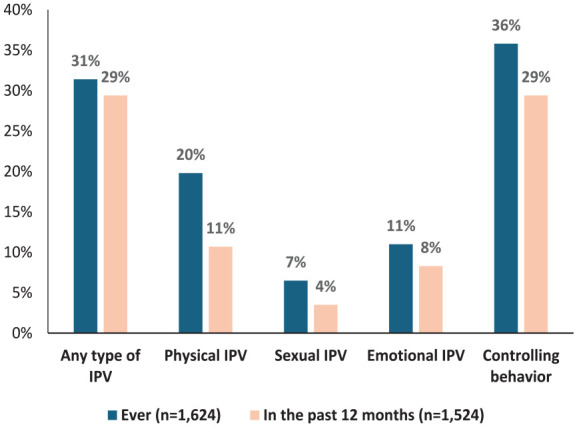
Type of IPV experienced by Nepalese women ever (*n* = 1624) and in the past 12 months (*n* = 1524). Note: Controlling behavior assessed among those with non-missing data for related survey questions, experiencing ever (*n* = 4160) and in the past 12 months (*n* = 4141).

As shown in Figure 1, out of the total surveyed women (*n* = 5178), 31.4% (*n* = 1624) reported having ever experienced some form of IPV. Specifically, 29.4% (*n* = 1524) women had experienced IPV in the last 12 months, with controlling behavior (29.4%, *n* = 1219) emerging as the most prevalent form, while sexual IPV (3.5%, *n* = 181) was the least frequently reported. Emotional IPV in the past 12 months was reported by 429 women (8.3%) and physical IPV by 551 women (10.7%).

**Figure 2.**
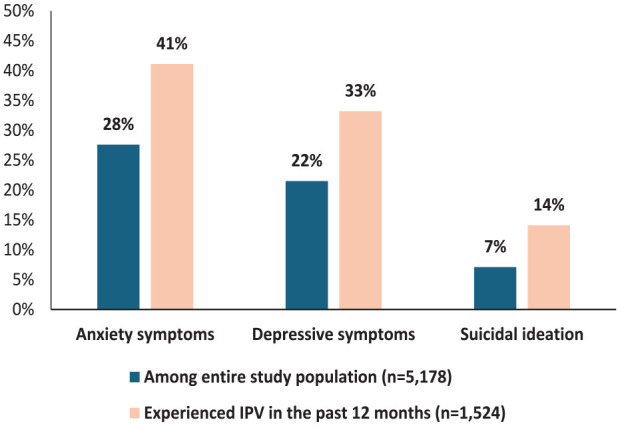
Experience of mental health problems over the last 2 weeks among the entire study population (*n* = 5178) and those experiencing IPV in the past 12 months (*n* = 1524). IPV: intimate partner violence.

The participants’ experience of mental health problems and help-seeking for IPV are summarized in Table 2 (unweighted numbers related to the participants’ experience of mental health problems and help-seeking for IPV are presented in Supplemental Appendix Table A3), while Table 3 presents the association between mental health problems and predictors, including sociodemographic and behavioral factors, and IPV among Nepalese women (all bivariate analyses for these factors, along with adjusted odds ratios (aORs) and *p*-values for statistically non-significant predictors are provided in Supplemental Appendix Tables A4.1 and A4.2). Mental health symptoms were significantly more prevalent among women who had experienced IPV. Figure 2 shows that out of the total surveyed women, 27.6% (*n* = 1427) reported anxiety symptoms, 21.5% (*n* = 1110) reported depressive symptoms, and 7.1% (*n* = 368) reported suicidal ideation within the past 2 weeks. Among women who had experienced IPV in the past 12 months, 41.1% (*n* = 627) reported experiencing anxiety, 33.2% (*n* = 506) reported symptoms of depression, and 14.1% (*n* = 215) reported suicidal ideation. In terms of the severity of mental health issues (Table 2), mild anxiety and mild depression were the most prevalent among both women who had experienced IPV in the past 12 months.

**Table 2.** Experience of mental health problems and help-seeking for IPV among women aged 15–49 (*n* = 5178).

Variable	*N*	%
Mental health problems		
Anxiety symptoms over the last 2 weeks (yes)	1427	27.6
Mild anxiety	1061	74.4
Moderate anxiety	286	20.0
Severe anxiety	80	5.6
Depressive symptoms over the last 2 weeks (yes)	1110	21.5
Mild depression	829	74.7
Moderate depression	196	17.6
Moderately severe depression	62	5.6
Severe depression	23	2.1
Suicidal ideation over the last 2 weeks (yes)	368	7.1
Mental health problems among those who have experienced IPV in the past 12 months		
Anxiety symptoms over the last 2 weeks (yes)	627	41.1
Mild anxiety	440	70.2
Moderate anxiety	136	21.7
Severe anxiety	51	8.1
Depressive symptoms over the last 2 weeks (yes)	506	33.2
Mild depression	350	69.2
Moderate depression	105	20.8
Moderately severe depression	33	6.5
Severe depression	18	3.5
Suicidal ideation over the last 2 weeks (yes)	215	14.1
Help-seeking for IPV^a^		
No	885	72.0
Yes	345	28.0
Informal help^b^	318	92.2
Formal help^c^	9	2.6
Both (informal and formal help)	18	5.2

All counts and percentages were calculated using sample weights. IPV: intimate partner violence.

a Out of those who had ever experienced physical or sexual IPV and were asked about help-seeking (*n* = 1230).

b Natal family, husband’s or partner’s family, partner, friends, neighbors, religious leaders, or others.

c Doctors, police, lawyers, or social work organizations.

**Table 3.** The association between mental health problems and predictors, including sociodemographic and behavioral factors, and IPV among Nepalese women.

Predictor	Anxiety (yes)				Depression (yes)				Suicidal ideation (yes)			
	Unadjusted OR (95% CI)	*p*-Value	aOR^a,*^ (95% CI)	*p*-Value	Unadjusted OR (95% CI)	*p*-Value	aOR^b,*^ (95% CI)	*p*-Value	Unadjusted OR (95% CI)	*p*-Value	aOR^c,*^ (95% CI)	*p*-Value
Frequency of partner being drunk												
Never	Reference				Reference				Reference			
Sometimes	1.55 (1.21–1.98)	<0.001	1.27 (0.97–1.65)	0.077	1.42 (1.09–1.86)	0.010	1.20 (0.90–1.61)	0.211	1.73 (1.10–2.74)	0.019	1.19 (0.75–1.90)	0.455
Often	4.14 (2.89–5.94)	<0.001	2.18 (1.46–3.25)	<0.001	3.60 (2.44–5.30)	<0.001	2.03 (1.32–3.14)	0.001	4.96 (3.08–7.98)	<0.001	1.96 (1.08–3.57)	0.027
Province of residence												
Bagmati	Reference				Reference				Reference			
Koshi	1.34 (0.98–1.84)	0.066	1.84 (1.13–3.00)	0.015	1.69 (1.20–2.36)	0.002	1.99 (1.26–3.15)	0.003	1.78 (1.07–2.95)	0.025	1.60 (0.72–3.58)	0.251
Madhesh	1.04 (0.73–1.50)	0.812	0.84 (0.45–1.55)	0.576	1.09 (0.73–1.63)	0.675	0.89 (0.49–1.61)	0.689	1.33 (0.75–2.35)	0.326	1.09 (0.47–2.53)	0.847
Gandaki	0.86 (0.60–1.23)	0.402	1.19 (0.78–1.83)	0.422	0.98 (0.66–1.45)	0.917	0.95 (0.59–1.52)	0.829	1.11 (0.59–2.08)	0.757	1.03 (0.50–2.12)	0.933
Lumbini	1.06 (0.76–1.49)	0.723	1.24 (0.77–2.00)	0.378	1.06 (0.74–1.53)	0.753	0.97 (0.61–1.55)	0.895	1.41 (0.82–2.42)	0.214	1.31 (0.62–2.75)	0.481
Karnali	1.66 (1.19–2.31)	0.003	2.31 (1.39–3.83)	0.001	1.94 (1.41–2.67)	<0.001	2.38 (1.48–3.84)	<0.001	2.62 (1.61–4.24)	<0.001	2.11 (1.00–4.42)	0.049
Province of residence												
Sudurpaschim	1.38 (0.98–1.94)	0.065	1.88 (1.12–3.17)	0.017	1.27 (0.90–1.79)	0.173	1.37 (0.84–2.23)	0.213	1.61 (0.96–2.70)	0.071	1.57 (0.68–3.63)	0.286
Experienced any type of IPV in the last 12 months (yes)	2.49 (2.10–2.96)	<0.001	1.52 (0.90–2.55)	0.118	2.51 (2.09–3.01)	<0.001	0.75 (0.45–1.25)	0.277	3.75 (2.81–5.00)	<0.001	0.95 (0.48–1.88)	0.890
Physical IPV (yes)	2.76 (2.20–3.46)	<0.001	1.06 (0.71–1.57)	0.790	2.94 (2.30–3.76)	<0.001	1.07 (0.72–1.60)	0.725	4.52 (3.25–6.28)	<0.001	1.55 (0.92–2.61)	0.100
Sexual IPV (yes)	6.06 (4.21–8.71)	<0.001	2.88 (1.67–4.95)	<0.001	4.70 (3.39–6.52)	<0.001	2.12 (1.32–3.41)	0.002	5.56 (3.62–8.53)	<0.001	1.63 (0.90–2.96)	0.108
Emotional IPV (yes)	4.97 (3.86–6.40)	<0.001	3.00 (2.08–4.31)	<0.001	4.83 (3.71–6.29)	<0.001	3.09 (2.19–4.37)	<0.001	5.16 (3.88–6.86)	<0.001	1.91 (1.20–3.04)	0.006
Controlling behavior (yes)	2.28 (1.90–2.74)	<0.001	1.03 (0.65–1.62)	0.896	2.42 (2.02–2.89)	<0.001	1.88 (1.21–2.92)	0.005	3.44 (2.56–4.63)	<0.001	2.24 (1.25–4.02)	0.007
Ever experienced any type of IPV (yes)^d^	2.73 (2.33–3.20)	<0.001	1.20 (0.78–1.86)	0.412	2.85 (2.39–3.39)	<0.001	0.89 (0.54–1.45)	0.634	4.57 (3.45–6.05)	<0.001	1.24 (0.61–2.52)	0.553
Physical IPV (yes)	2.66 (2.24–3.16)	<0.001	1.01 (0.69–1.47)	0.971	2.75 (2.26–3.35)	<0.001	1.18 (0.79–1.76)	0.424	4.14 (3.15–5.43)	<0.001	1.27 (0.72–2.23)	0.410
Sexual IPV (yes)	4.70 (3.60–6.14)	<0.001	2.21 (1.44–3.39)	<0.001	4.03 (3.17–5.13)	<0.001	1.61 (1.09–2.37)	0.017	5.57 (4.12–7.53)	<0.001	1.68 (1.08–2.61)	0.021
Emotional IPV (yes)	4.18 (3.36–5.20)	<0.001	2.22 (1.58–3.12)	<0.001	4.51 (3.50–5.80)	<0.001	2.65 (1.82–3.87)	<0.001	5.73 (4.30–7.64)	<0.001	2.30 (1.42–3.74)	0.001
Controlling behavior (yes)	2.36 (1.98–2.82)	<0.001	1.31 (0.93–1.86)	0.123	2.58 (2.17–3.06)	<0.001	1.56 (1.05–2.32)	0.028	3.91 (2.89–5.29)	<0.001	1.94 (1.04–3.60)	0.036

All values were calculated using sample weights. All bivariate analyses for sociodemographic and behavioral factors are presented in Supplemental Appendix Tables A4.1 and A4.2. OR: odds ratio; CI: confidence interval; aOR: adjusted odds ratio; IPV: intimate partner violence.

a Adjusted for: age, education, employment, province, marital status, frequency of partner being drunk, and woman drinking.

b Adjusted for education, province, type of residence (urban/rural), ethnicity, marital status, household wealth, and frequency of partner being drunk.

c Adjusted for education, type of residence (urban/rural), marital status, household wealth, and frequency of partner being drunk.

d A separate model was created to analyze associations with this variable, adjusted for the same confounders as in other models (see a, b, c).

* aORs and related *p*-values for statistically non-significant predictors are presented in Supplemental Appendix Tables A4.1 and A4.2.

The odds of experiencing mental health problems were notably higher among women who had experienced sexual or emotional IPV, as well as controlling behavior, compared to those who had not experienced IPV (Table 3). Specifically, women who reported sexual IPV in the past 12 months had nearly three times higher odds of anxiety (aOR 2.88, 95% CI 1.67–4.95), and the odds of depression were twice as high (aOR 2.12, 95% CI 1.32–3.41), while the odds of suicidal ideation were nearly doubled (aOR 1.63, 95% CI 0.90–2.96) in the same group. Experiencing emotional IPV was associated with a three-fold increase in the odds of anxiety (aOR 3.00, 95% CI 2.08–4.31) and depression (aOR 3.09, 95% CI 2.19–4.37), while the odds for suicidal ideation were nearly twice as high (aOR 1.91, 95% CI 1.20–3.04). Similarly, controlling behavior approximately doubled the odds of depression (aOR 1.88, 95% CI 1.21–2.92) and suicidal ideation (aOR 2.24, 95% CI 1.25–4.02).

Residing in Karnali province more than doubled the odds for anxiety (aOR 2.31, 95% CI 1.39–3.83), depression (aOR 2.38, 95% CI 1.48–3.84), and suicidal ideation (aOR 2.11, 95% CI 1.00–4.42) compared to living in Bagmati. When the partner was frequently drunk, the odds of anxiety (aOR 2.18, 95% CI 1.46–3.25), depression (aOR 2.03, 95% CI 1.32–3.14) and suicidal ideation (aOR 1.96, 95% CI 1.08–3.57) were twice as common.

Regarding help-seeking behavior to address IPV, among the 1230 women surveyed, the majority (72.0%) had not sought help to stop the violence (Table 2). Of the 28.0% (*n* = 350) who sought help, 318 women (92.2%) sought informal help, with only 9 women (2.6%) seeking formal help. Table 4 presents the association between sociodemographic and behavioral factors and seeking help to stop the violence among women who have experienced physical and sexual violence (all bivariate analyses for these factors along with aORs and *p*-values for statistically non-significant predictors are presented in Supplemental Appendix Table A5). If the partner was frequently drunk, the odds of seeking help to stop the violence doubled (aOR 2.17, 95% CI 1.17–4.02). Conversely, for women residing in Sudurpaschim province, the odds of seeking help to stop the violence were reduced by 61% (aOR 0.39, 95% CI 0.17–0.89).

**Table 4.** The association between sociodemographic and behavioral factors and seeking help to stop the violence among women who have experienced physical or sexual IPV (*n* = 1230).

Predictor	Have sought help for IPV (yes)			
	Unadjusted OR (95% CI)	*p*-Value	aOR^a^ (95% CI)	*p*-Value
Frequency of partner being drunk				
Never	Reference		Reference	
Sometimes	1.14 (0.63–2.05)	0.659	1.01 (0.57–1.77)	0.973
Often	2.28 (1.21–4.29)	0.011	2.17 (1.17–4.02)	0.014
Province of residence				
Bagmati	Reference		Reference	
Koshi	0.96 (0.49)	0.915	1.22 (0.59–2.55)	0.593
Madhesh	0.70 (0.36–1.36)	0.290	1.01 (0.44–2.34)	0.975
Gandaki	0.77 (0.38–1.55)	0.458	0.78 (0.34–1.79)	0.559
Lumbini	0.83 (0.43–1.59)	0.571	1.05 (0.50–2.20)	0.902
Karnali	0.79 (0.39–1.60)	0.507	0.56 (0.24–1.32)	0.185
Sudurpaschim	0.40 (0.19–0.84)	0.015	0.39 (0.17–0.89)	0.025

All values were calculated using sample weights. All bivariate analysis for sociodemographic and behavioral factors are presented in Supplemental Appendix Table A5. IPV: intimate partner violence; aOR: adjusted odds ratio.

a Adjusted for: employment, province, ethnicity, marital status, frequency of partner being drunk, and frequency of woman drinking. aORs and related *p*-values for statistically non-significant predictors are presented in Supplemental Appendix Table A5.

**Figure 3.**
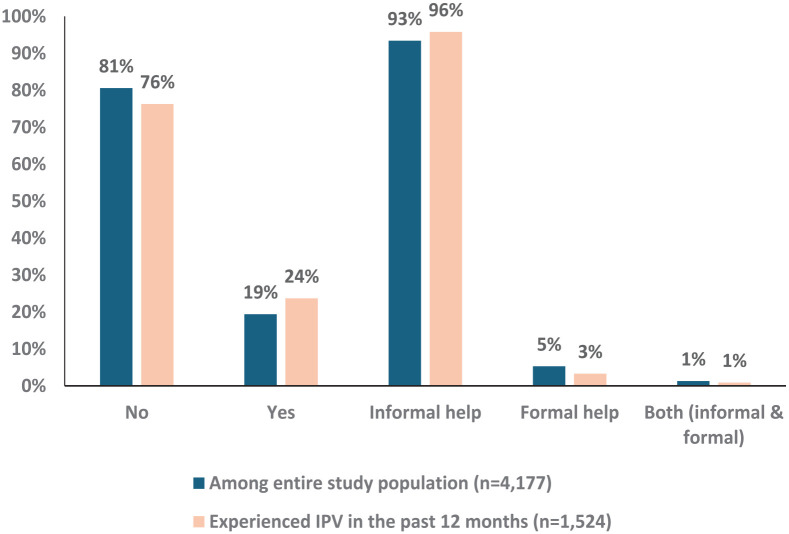
Help-seeking for mental health problems among the entire study population with non-missing data for related survey questions (*n* = 4177) and women experiencing IPV in the past 12 months (*n* = 1524). IPV: intimate partner violence.

Lastly, among the 4194 women asked about seeking help for mental health issues, 19.4% (*n* = 812) reported doing so (Figure 3). Of those, 93.4% (*n* = 759) sought informal help, 5.3% (*n* = 43) sought formal help, and 1.3% (*n* = 10) sought help from both informal and formal sources. Among women who reported experiencing IPV in the past 12 months and who were queried about seeking help for mental health issues (*n* = 1392), the rate was slightly higher, with 23.7% (*n* = 330) seeking help. However, among these women, seeking informal help was more common, with the vast majority (95.8%, *n* = 316) opting for this approach.

The odds of seeking help for mental health problems were 1.6 times higher among women experiencing anxiety (aOR 1.67, 95% CI 1.14–2.45) or depression (aOR 1.63, 95% CI 1.03–2.57) and IPV in the past 12 months (Table 5, and all bivariate analyses along with aORs and related *p*-values in Supplemental Appendix Table A6). Additionally, women residing in Sudurpaschim province had twice the odds of seeking help for mental health problems (aOR 2.09, 95% CI 1.11–3.96), while living in a rich household more than doubled the odds (aOR 2.24, 95% CI 1.39–3.61). Ethnicity, marital status, and frequency of partner being drunk, or woman drinking were not found to be significantly associated with seeking help for mental health issues.

**Table 5.** Factors associated with help-seeking for mental health problems among Nepalese women who have experienced IPV in the past 12 months (*n* = 1524).

Predictor	Have sought help for mental health problems (yes)			
	Unadjusted OR (95% CI)	*p*-Value	aOR^a^ (95% CI)	*p*-Value
Household wealth				
Poor (0%–40%)	Reference		Reference	
Middle (40%–80%)	1.34 (1.07–1.69)	0.012	1.23 (0.89–1.70)	0.205
Rich (80%–100%)	1.19 (0.89–1.56)	0.229	2.24 (1.39–3.61)	0.001
Province of residence				
Bagmati	Reference		Reference	
Koshi	1.52 (0.97–2.38)	0.066	1.64 (0.93–2.87)	0.084
Madhesh	1.33 (0.81–2.19)	0.254	1.71 (0.87–3.36)	0.121
Gandaki	1.08 (0.63–1.86)	0.783	1.46 (0.77–2.76)	0.247
Lumbini	1.27 (0.79–2.05)	0.318	1.75 (0.99–3.10)	0.055
Karnali	1.20 (0.79–1.82)	0.392	1.46 (0.82–2.59)	0.197
Sudurpaschim	1.43 (0.88–2.32)	0.144	2.09 (1.11–3.96)	0.023
Anxiety symptoms (yes)^b^	3.63 (2.90–4.56)	<0.001	1.67 (1.14–2.45)	0.008
Depressive symptoms (yes)	3.93 (3.05–5.05)	<0.001	1.63 (1.03–2.57)	0.036
Suicidal ideation (yes)^b^	3.59 (2.52–5.11)	<0.001	1.20 (0.73–1.94)	0.463

All values were calculated using sample weights. All bivariate analysis for sociodemographic and behavioral factors are presented in Supplemental Appendix Table A6. IPV: intimate partner violence; aOR: adjusted odds ratio.

a Adjusted for: province, ethnicity, marital status, household wealth, frequency of partner being drunk, frequency of woman drinking and anxiety, depressive symptoms, and suicidal ideation among women experiencing IPV in the past 12 months. aORs and related *p*-values for statistically non-significant predictors are presented in Supplemental Appendix Table A6.

b Among women experiencing IPV in the past 12 months (*n* = 1524).

## Discussion

This is the first nation-wide study from Nepal on IPV and mental health that is representative for all women of reproductive age (15–49), both married and unmarried. It shows that IPV is highly prevalent in Nepal with 31.4% of women aged 15–49 having faced some form of IPV in their lifetime, and almost all of these (29.4%) had experienced IPV in the past 12 months. Not surprisingly, IPV was significantly associated with mental health problems. Emotional and sexual IPV, as well as controlling behavior, were linked with depression. Emotional and sexual IPV also increased the likelihood of anxiety, while emotional IPV and controlling behavior heightened the likelihood of suicidal ideation. Additionally, help-seeking rates for both IPV and mental health problems were low, with almost all of those seeking help opting for informal sources.

These findings exceed the latest WHO estimates, which have suggested that 27% of Nepalese women aged 15 years and older, ever been married or partnered, have encountered IPV at least once in their lifetime, with 11% experiencing IPV in the past 12 months.^4^ However, a crucial difference is that WHOs estimates only cover sexual and/or physical IPV, unlike this study, which includes more forms of violence: physical, sexual, and emotional IPV, as well as controlling behavior.

Notably, a recent study conducted by Bhatt et al. during the COVID-19 pandemic revealed an even higher IPV prevalence, nearly 53% among married Nepalese women, reporting exposure to behavioral control, and economic, emotional, physical, and sexual violence.^5^ Several countries observed increased levels of IPV during pandemic lockdowns, partly explained by women’s isolation at home, financial and emotional household stress and reduced access to informal and formal support structures.^22^ These factors, the inclusion of economic violence and the use of purposive and snowball sampling techniques, may explain the higher prevalence rates observed by Bhatt et al. compared to the NDHS.

Controlling behavior (28.8%) emerged as the most prevalent form of IPV, while emotional (11%) and sexual IPV (6.5%) were reported less frequently. This aligns with Bhatt et al. who also found that behavioral control (and economic violence, not assessed by the NDHS) were the most prevalent types of IPV among Nepalese women, and sexual violence (14%) being the least common.^5^ Although sexual violence may indeed be less frequently reported, it is likely underreported, much like IPV in general, due to the stigma associated with it. In conservative settings such as Nepal, cultural norms and discomfort with discussing sexual matters may further limit disclosure.

This study reveals a clear association between experiencing IPV and a higher prevalence of mental health problems. Among women who had experienced IPV in the past 12 months, the prevalence of anxiety symptoms was 13.5% higher, depressive symptoms were 11.7% higher, and suicidal ideation was 7.6% higher compared to the entire study population. Since the 2022 NDHS represents Nepal’s first assessment of IPV and its mental health consequences at the national level, there are no prior Nepalese studies available for direct comparison. Nonetheless, global research consistently highlights IPV as a significant factor in mental health issues.^1^ Previous studies have shown correlations between IPV and various mental health issues, including anxiety, depression, and suicidal ideation,^11–13^ among others not specifically examined in this study.

Sexual and emotional IPV, along with controlling behavior, came out as significant risk factors for poor mental health, in line with previous research.^1,40^ Women who had experienced controlling behavior in the past year had the highest odds of suicidal ideation. Among all IPV survivors, those who faced emotional IPV had the highest prevalence of suicidal ideation. This highlights the profound psychological impact of these forms of abuse, underscoring the urgent need for support and intervention for individuals experiencing controlling behavior and emotional IPV.

There was no clear evidence indicating a higher prevalence of anxiety, depression, or suicidal ideation among women experiencing physical IPV. This stands in contrast with the findings of the systematic review by White et al.^12^ In their review, experiences of physical IPV were linked with significantly higher odds of anxiety, depression, and suicidal ideation.^12^ Although a slight increase in mental health problems was observed in the current study (Supplemental Appendix Table A4), these differences were not statistically significant. Given that the systematic review by White et al. mainly included original studies conducted in the United States and other high-income countries,^12^ the difference in study findings could be attributed to cultural disparities and prevailing gender norms. This includes the acceptability of physical abuse within intimate relationships, which may vary between high-income countries and Nepal. In Nepal, physical IPV is often seen as a normal aspect of women’s lives, with traditional gender roles being deeply rooted.^41^ On the other hand, a Sri Lankan study exploring the link between suicidal ideation and domestic violence (including emotional, sexual, and physical violence) found that the association was strongest with physical violence, suggesting that this connection requires further investigation.^42^

Somewhat surprisingly, among all the sociodemographic and behavioral factors investigated, only the province of residence and the frequency of a partner being drunk were statistically significantly associated with increased odds of mental health problems. Previous studies have shown that harmful alcohol consumption is a risk factor for IPV, which likely explains the higher odds of mental health issues in these cases.^8,43,44^ Evidence-based interventions, such as the Common Elements Treatment Approach, have been found effective and could be implemented in Nepal to address both women’s experiences of IPV and their partner’s alcohol misuse.^45^ Living in Karnali province more than doubled the odds for anxiety, depression, and suicidal ideation compared to residing in Bagmati. This disparity may be attributed to several factors, including Karnali province’s status as having Nepal’s second lowest human development index score, indicating challenges in achieving long-term progress in health, education, and standard of living.^46^ Moreover, it has the highest level of gender inequality, as reflected by the gender inequality index, which assesses reproductive health, empowerment, and economic activity.^46^

This study clearly indicates that the majority of women (72.0%) experiencing IPV do not seek help to escape or to stop the violence. Notably, this study solely focused on help-seeking behavior for women who had encountered physical or sexual IPV, thereby excluding survivors of emotional IPV and the most prevalent form of IPV, controlling behavior. However, despite these limitations, the rate of Nepalese women seeking help is still higher than previous studies in similar settings. In Bhatt et al.’s study conducted during the COVID-19 lockdowns, only 14% of Nepalese women facing IPV sought help, possibly associated with reduced access to health services.^5^ However, an Indian cross-sectional study pre-pandemic, reported a similarly low figure: only 13.8% of women who experienced physical, sexual, or emotional IPV had sought help to stop the violence.^47^ Reasons for slightly higher help-seeking behaviors in our nation-representative study remain unclear but could be explained to a fairly recent scale-up of one-stop crisis centers across Nepal which may have increased societal awareness. Additionally, we noticed that the likelihood of seeking help for mental health issues was slightly higher among women who had experienced IPV in the past 12 months (23.7%) compared to the entire study population (19.4%), likely reflecting the higher prevalence of mental health problems among those who have experienced violence.

For women residing in Sudurpaschim province, the odds of seeking help to stop the violence were 61% lower than in Bagmati (aOR 0.39, 95% CI 0.17–0.89). This province faces challenges such as the second-highest unemployment rate in the country, below-average literacy rates, and per capita incomes, all factors that may influence the probability of seeking help.^46^ Despite the decreased odds of seeking help to address violence among women in Sudurpaschim, their likelihood of seeking help for mental health problems was twice that of Bagmati (aOR 2.09, 95% CI 1.11–3.96). This suggests that it may be easier or more socially acceptable to seek help for mental health issues than for IPV. It may also indicate that IPV is perceived as a more private matter and/or carries a greater stigma or has more legal and social implications than seeking help for mental health problems.

The low rates of help-seeking behavior found for both IPV and mental health problems are consistent with previous research.^47,48^ Low help-seeking rates for mental health issues specifically can be partly attributed to the limited availability of mental health care facilities and professionals across the country.^23,26,49^ However, societal beliefs, traditions, and the stigma associated with mental disorders contribute as a significant factor to the treatment gap.^26^ This prevailing stigma creates a double burden for those affected by both IPV and mental health issues. Moreover, the study aligns with previous research, indicating that women are significantly more inclined to seek help from informal rather than formal sources.^5,47^ Among women reporting IPV, this preference was slightly more pronounced, with 95.8% choosing informal help for mental health problems compared to 93.4% among the overall study population. These findings highlight the importance of improving access to formal support services for women facing IPV and mental health problems.

This study contributes to the growing body of evidence that IPV is associated with serious mental health problems and highlights the extent to which many women manage IPV and mental health issues without any support from the health system. These findings underscore the importance of early detection of IPV both within healthcare facilities and at the community level, as well as the need to connect IPV survivors with the available support services. Furthermore, this study emphasizes the urgent need for strong policy and health system responses to tackle these interrelated challenges effectively and increase public awareness of IPV.

Given the substantial mental health consequences associated with emotional IPV and controlling behavior, it is concerning that these forms of IPV often receive less attention and are underexamined and underrecognized. For instance, the WHOs global, regional, and national prevalence estimates primarily focus on physical and sexual IPV.^4^ Without acknowledgment and measurement of emotional IPV and controlling behavior, the health system cannot adapt to meet the needs of these women. Moreover, this oversight potentially diminishes their likelihood of seeking help and mitigates their overall health outcomes. Therefore, enhancing surveillance systems to monitor IPV is important, enabling timely interventions.

Despite notable progress in human development, gender inequality persists in Nepal,^5,46,47^ serving as a significant risk factor for IPV^10^ and potentially hindering help-seeking behaviors. The reluctance to report IPV is influenced by stigma attached to it and cultural norms dictating women’s roles,^50^ underscoring the need for cultural change. At the societal level, it is crucial to condemn IPV unequivocally and hold perpetrators accountable through prosecution, thereby empowering survivors to seek the help they require. Furthermore, interventions that involve men in efforts to prevent IPV have been shown to be more effective than those targeting women only.^51^

One-stop crisis management centers are intended to provide robust support to IPV survivors^23^ free of charge,^21^ but the varying availability and quality of these services in Nepal^21^ may explain why women hesitate to seek help from them. Additionally, evidence from the region highlights a lack of support from authorities, including healthcare workers and police, as IPV is often perceived as a private matter in which outsiders should not intervene.^52^ Authorities are also frequently viewed as socially inappropriate or unapproachable.^53^ This lack of institutional support diminishes trust in both the medical and legal systems, discouraging victims from seeking help and increasing the risk of continued violence. While previous studies have identified various factors and barriers to seeking help for IPV, further research including qualitative methods, is needed to assess and understand why, despite the availability of some formal services at no cost or minimal cost from health facilities and other formal sectors, violence survivors rarely utilize such services in the context of Nepal. Understanding the underlying reasons for the low uptake of formal support services is crucial for improving the accessibility and utilization of available formal support services by violence survivors in Nepal and other low- and middle-income countries.

Previous work indicates that women normally prefer healthcare professionals to initiate discussions about IPV,^50^ highlighting the importance of these being knowledgeable about IPV, proactively inquire about IPV experiences, as well as being prepared to identify and assist IPV survivors. Given that pregnancy is a time when most women seek healthcare services, antenatal care professionals should routinely inquire about women’s experiences with IPV. This is especially important considering that pregnancy itself as a risk factor for IPV.^8^ However, only 28% of health facilities in Nepal have trained their staff to manage violence against women, indicating the need for comprehensive training of healthcare professionals in violence management skills across all health system levels.^23^ Such training would significantly enhance their ability to offer appropriate support. Furthermore, a lack of knowledge among healthcare professionals may decrease the trust of IPV survivors in the healthcare system, diminishing their likelihood of seeking help. However, further research is necessary to understand the reasons behind women’s lack of confidence in the healthcare system and their reluctance to seek help from formal channels.

In addition to formal support systems, informal networks should be equipped with the necessary knowledge to assist women facing IPV, enabling them to guide these women toward formal help. The findings of this study underscore the importance of informal support in the help-seeking process and underscore the necessity for interventions that involve survivors’ informal networks in IPV outreach and response efforts. Without training informal sources, IPV survivors will not receive the support they need. Additionally, it is important to educate peers and community members about mental health problems, including their signs and symptoms, and to guide how to offer support, where to seek help, and how to access support services. According to a systematic review by Ogbe et al., IPV interventions that incorporate advocacy with a focus on community networks significantly improve mental health outcomes and access to support services.^54^ One effective approach is to train IPV survivors as counselors and mentors for other survivors, enabling them to assist abused women in accessing services, guiding them through safety planning, and enhancing their physical and psychological health.^54^ This approach could be effectively implemented in Nepal. Furthermore, community mobilization interventions like SASA! could be a powerful tool in reducing the overall prevalence of IPV.^55^ Such programs raise awareness within communities and foster support to prevent violence against women.^55^

These study findings can provide valuable data for policymakers to formulate evidence-based policies addressing IPV and its mental health impacts. Policies that promote an integrated approach, such as the implementation of not only institutional services provided by public health facilities or law enforcement agencies but also community-based interventions for prevention, treatment, care, and support services for violence survivors, would be effective in eliminating violence against women. Additionally, addressing the significant gap in policy implementation is crucial, as current policies are not being effectively enforced. Without this, services for IPV will remain underutilized, and perpetrators may continue to go unpunished. Conducting regular assessments is essential to evaluate the effectiveness of implemented policies and programs, ensuring continuous improvement. Furthermore, the results justify allocating more resources toward IPV support programs and mental health services.

### Strengths and limitations

The 2022 NDHS represents the first nationwide and provincial assessment undertaken by the country concerning IPV and its ramifications on mental health. Supported by the DHS program, known for its collaboration with governments to collect and disseminate data regarding people, their health, and health systems, this partnership ensures quality and standardizations at both regional and global levels.^29^ Consequently, it enables the comparability of results across various countries. However, the 2022 NDHS marks the first time of collecting data on IPV from never-married women who reported either current or former intimate partnerships, not solely from ever-married women. Consequently, only the estimates specifically outlined for ever-married women and women cohabiting with a partner should be compared with similar data from previous surveys.

This population-based study benefits from its robust sample size of 5178 women. With such a large sample, the study supports detailed and comprehensive analyses while maintaining acceptable levels of sampling error. Furthermore, the sampling frame used in this study comprises a comprehensive list of all sampling units covering the entire target population. Employing scientific probability sampling techniques, the study minimizes selection bias. Standardized measures of anxiety and depressive symptoms enhance the reliability of the findings. Moreover, the study’s findings are applicable beyond its specific context, offering insights relevant to other low- and middle-income countries.

Since the analysis is based on cross-sectional data, causal relationships cannot be established. The assessment of anxiety and depressive symptoms relied on widely accepted and validated measures; however, it did not include standardized diagnostic interviews. Consequently, some women identified as having clinically significant symptoms may not meet the criteria for a mental health disorder during a psychiatric evaluation, while others scoring below clinical thresholds may be misclassified. Although these classification biases are unlikely to substantially impact the results, they should be taken into account when comparing findings across studies.

The survey specifically asked ever-married women about violence perpetrated by current or former husbands only, excluding incidents involving potential previous intimate partners such as boyfriends. This approach may have overlooked instances of IPV experienced by other partners, potentially influencing the findings. Lastly, the 2022 NDHS was conducted in the COVID-19 pandemic afterthought, which previous studies suggest may have not only increased the occurrence of IPV but also posed additional challenges for women seeking help for both IPV and mental health issues.

## Conclusions

This cross-sectional study provides new evidence on a high prevalence of IPV and associated mental health problems, including anxiety, depression, and suicidal ideation among Nepalese women aged 15–49 experiencing IPV. It underscores the pressing need for robust health system responses to address these interrelated challenges. Moreover, the study reveals that Nepalese women rarely seek help to stop the violence or for mental health problems. When they do seek help, the majority opt for informal sources. Hence, informal support networks play a crucial role in providing avenues for aid to women grappling with IPV and mental health problems. Training these informal sources is vital to effectively reach IPV survivors and offer them the support they require. Despite ongoing efforts through policy and programmatic measures to combat IPV and address mental health problems, further action is urgently needed. This entails conducting comprehensive research to understand the factors contributing to the underutilization of formal help services and using this knowledge to develop programs and initiatives aimed at reaching marginalized and vulnerable groups and increasing their access to health services.

## Supplemental Material

sj-docx-1-whe-10.1177_17455057251326416 – Supplemental material for Burden of intimate partner violence, mental health issues, and help-seeking behaviors among women in Nepal Supplemental material, sj-docx-1-whe-10.1177_17455057251326416 for Burden of intimate partner violence, mental health issues, and help-seeking behaviors among women in Nepal by Monna Kurvinen, Anna Mia Ekström and Keshab Deuba in Women’s Health

sj-docx-2-whe-10.1177_17455057251326416 – Supplemental material for Burden of intimate partner violence, mental health issues, and help-seeking behaviors among women in Nepal Supplemental material, sj-docx-2-whe-10.1177_17455057251326416 for Burden of intimate partner violence, mental health issues, and help-seeking behaviors among women in Nepal by Monna Kurvinen, Anna Mia Ekström and Keshab Deuba in Women’s Health

## Declarations

**Ethics approval and consent to participate:** The survey protocol underwent review by both the Nepal Health Research Council and the ICF Institutional Review Board.^28^ The authors of this study also obtained approval from ICF International to analyze the data. During the primary data collection, written consent from the household head was required for conducting the interviews.^28^ The 2022 NDHS received ethical approval from the Ethical Review Board of the Nepal Health Research Council (Reference number: 494-2021).

**Consent for publication:** Not applicable.

**Availability of data and materials:** The datasets used and/or analyzed during the study are available from the ICF International on reasonable request.

## References

[1] Oram S Fisher HL Minnis H , et al. The Lancet Psychiatry Commission on intimate partner violence and mental health: advancing mental health services, research, and policy. Lancet Psychiatry 2022; 9(6): 487–524. 35569504 10.1016/S2215-0366(22)00008-6

[2] United Nations. Intensification of efforts to prevent and eliminate all forms of violence against women and girls. Report (A/77/302), United Nations, August 2022.

[3] World Health Organization. Violence info—intimate partner violence, http://apps.who.int/violence-info/intimate-partner-violence (2022, accessed 13 February 2024).

[4] World Health Organization. Violence against women prevalence estimates, 2018. Global, regional and national prevalence estimates for intimate partner violence against women and global and regional prevalence estimates for non-partner sexual violence against women. Report. Geneva: World Health Organization, 2021.

[5] Bhatt B Bhatt N Karki A , et al. Intimate partner violence against married women of reproductive age in Nepal during the COVID-19 pandemic. Heliyon 2023; 9(9): e20117. 10.1016/j.heliyon.2023.e20117 PMC10559861 37809852

[6] Kyle J. Intimate partner violence. Med Clin North Am 2023; 107(2): 385–395. 36759104 10.1016/j.mcna.2022.10.012

[7] World Health Organization. Violence against women, https://www.who.int/news-room/fact-sheets/detail/violence-against-women (2024, accessed 12 February 2024).

[bibr8-17455057251326416] Yakubovich AR Stöckl H Murray J , et al. Risk and protective factors for intimate partner violence against women: systematic review and meta-analyses of prospective–longitudinal studies. Am J Public Health 2018; 108(7): e1–11. 10.2105/AJPH.2018.304428 PMC5993370 29771615

[bibr9-17455057251326416] Clark CJ Cheong YF Gupta J , et al. Intimate partner violence in Nepal: Latent patterns and association with depressive symptoms. SSM Popul Health 2019; 9: 100481. 31993482 10.1016/j.ssmph.2019.100481 PMC6978479

[10] Dills J Jones K Brown P. Continuing the dialogue: learning from the past and looking to the future of intimate partner violence and sexual violence prevention. Atlanta, GA: National Center for Injury Prevention and Control, Centers for Disease Control and Prevention, 2019.

[11] Dillon G Hussain R Loxton D , et al. Mental and physical health and intimate partner violence against women: a review of the literature. Int J Fam Med 2013; 23; 1–15. 10.1155/2013/313909 PMC3566605 23431441

[bibr12-17455057251326416] White SJ Sin J Sweeney A , et al. Global prevalence and mental health outcomes of intimate partner violence among women: a systematic review and meta-analysis. Trauma Violence Abuse 2024; 25(1): 494–511. 36825800 10.1177/15248380231155529 PMC10666489

[13] Pico-Alfonso MA Garcia-Linares MI Celda-Navarro N , et al. The impact of physical, psychological, and sexual intimate male partner violence on women’s mental health: depressive symptoms, posttraumatic stress disorder, state anxiety, and suicide. J Womens Health (Larchmt) 2006; 15(5): 599–611. 16796487 10.1089/jwh.2006.15.599

[14] Stewart DE Vigod SN. Mental health aspects of intimate partner violence. Psychiatr Clin North Am 2017; 40(2): 321–334. 28477656 10.1016/j.psc.2017.01.009

[15] Bandara P Page A Senarathna L , et al. Clinical and psychosocial factors associated with domestic violence among men and women in Kandy, Sri Lanka. PLoS Glob Public Health 2022; 2(4): e0000129. 10.1371/journal.pgph.0000129 PMC10021245 36962126

[16] American Psychological Association. APA Dictionary of Psychology, https://dictionary.apa.org/ (2018, accessed 13 February 2024).

[17] Cornally N McCarthy G. Help-seeking behaviour: a concept analysis. Int J Nurs Pract 2011; 17(3): 280–288. 21605269 10.1111/j.1440-172X.2011.01936.x

[18] Wright EN Anderson J Phillips K , et al. Help-seeking and barriers to care in intimate partner sexual violence: a systematic review. Trauma Violence Abuse 2022; 23(5): 1510–1528. 33685295 10.1177/1524838021998305

[19] Beeble ML Bybee D Sullivan CM , et al. Main, mediating, and moderating effects of social support on the well-being of survivors of intimate partner violence across 2 years. J Consult Clin Psychol 2009; 77(4): 718–729. 19634964 10.1037/a0016140

[20] García-Moreno C Hegarty K d’Oliveira AFL , et al. The health-systems response to violence against women. Lancet 2015; 385(9977): 1567–1579. 25467583 10.1016/S0140-6736(14)61837-7

[21] United Nations Nepal. Responding to domestic violence: a resource guide for UN personnel in Nepal, https://nepal.un.org/en/125900-responding-domestic-violence-resource-guide-un-personnel-nepal (2021, accessed 9 March 2024).

[22] Robinson SR Ravi K Voth Schrag RJ. A systematic review of barriers to formal help seeking for adult survivors of IPV in the United States, 2005–2019. Trauma Violence Abuse 2021; 22(5): 1279–1295. 32266870 10.1177/1524838020916254

[23] Deuba K Shrestha R Koju R , et al. Assessing the Nepalese health system’s readiness to manage gender-based violence and deliver psychosocial counselling. Health Policy Plan 2024; 39(2): 198–212. 38300229 10.1093/heapol/czae003 PMC10883662

[24] Rai Y Gurung D Gautam K. Insight and challenges: mental health services in Nepal. BJPsych Int 2021; 18(2): E5. 10.1192/bji.2020.58 PMC8274424 34287402

[bibr25-17455057251326416] Gupta AK Joshi S Kafle B , et al. Pathways to mental health care in Nepal: a 14-center nationwide study. Int J Ment Health Syst 2021; 15(1): 1–9. 34930398 10.1186/s13033-021-00509-4 PMC8685796

[26] Nepal Health Research Council. Report of National Mental Health Survey 2020. Government of Nepal, 2021.

[27] Von Elm E Altman DG Egger M , et al. The strengthening the reporting of observational studies in epidemiology (STROBE) statement: guidelines for reporting observational studies. Lancet 2007;370(9596): 1453–1457. 18064739 10.1016/S0140-6736(07)61602-X

[28] Ministry of Health and Population, New ERA, The DHS Program, and ICF. Nepal Demographic and Health Survey 2022, Ministry of Health and Population, Nepal, 2023.

[29] U.S. Agency for International Development. The Demographic and Health Surveys Program, https://www.usaid.gov/global-health/demographic-and-health-surveys-program (accessed 13 February 2024).

[30] Government of Nepal, Office of the Prime Minister and Council of Ministers, and National Statistics Office. National population and housing census 2021. Nepal: National Statistics Office, 2021.

[31] Human Rights Watch. Between a rock and a hard place: civilians struggle to survive in Nepal’s Civil War, https://www.hrw.org/reports/2004/nepal1004/ (2004, accessed 19 October 2024).

[32] World Health Organization. Putting women first: ethical and safety recommendations for research on domestic violence against women, https://www.who.int/publications-detail-redirect/WHO-FCH-GWH-01.1 (2001, accessed 18 February 2024).

[33] Spitzer RL Kroenke K Williams JBW , et al. A brief measure for assessing generalized anxiety disorder: The GAD-7. Arch Intern Med 2006; 166(10): 1092–1097. 16717171 10.1001/archinte.166.10.1092

[34] The Demographic and Health Surveys Program. Mental health module—model individual questionnaire. https://www.dhsprogram.com/publications/publication-DHSQM-DHS-Questionnaires-and-Manuals.cfm (2022, accessed 18 February 2024).

[35] Kroenke K Spitzer RL Williams JBW . The PHQ-9: validity of a brief depression severity measure. J Gen Intern Med 2001; 16(9): 606–613. 11556941 10.1046/j.1525-1497.2001.016009606.x PMC1495268

[36] Chowdhury MZI Turin TC . Variable selection strategies and its importance in clinical prediction modelling. Fam Med Community Health 2020; 8(1): e000262. 10.1136/fmch-2019-000262 PMC7032893 32148735

[37] Zhang Z. Model building strategy for logistic regression: purposeful selection. Ann Transl Med 2016; 4(6): 111. 27127764 10.21037/atm.2016.02.15 PMC4828741

[38] Peduzzi P Concato J Kemper E , et al. A simulation study of the number of events per variable in logistic regression analysis. J Clin Epidemiol 1996; 49(12): 1373–1379. 8970487 10.1016/s0895-4356(96)00236-3

[39] Vittinghoff E McCulloch CE. Relaxing the rule of ten events per variable in logistic and Cox regression. Am J Epidemiol 2007; 165(6): 710–718. 17182981 10.1093/aje/kwk052

[40] Clark CJ Ferguson G Shrestha B , et al. Mixed methods assessment of women’s risk of intimate partner violence in Nepal. BMC Womens Health 2019; 19(1): 20. 30691430 10.1186/s12905-019-0715-4 PMC6350343

[41] Colombini M Shrestha S Pereira S , et al. Comparing health systems readiness for integrating domestic violence services in Brazil, occupied Palestinian Territories, Nepal and Sri Lanka. Health Policy Plan 2024; 39(6): 552–563. 38758072 10.1093/heapol/czae032 PMC11145909

[42] Bandara P Page A Senarathna L , et al. Domestic violence and self-poisoning in Sri Lanka. Psychol Med 2022; 52(6): 1183–1191. 32912344 10.1017/S0033291720002986 PMC7612699

[43] Jayasuriya V Wijewardena K Axemo P. Intimate partner violence against women in the capital province of Sri Lanka: prevalence, risk factors, and help seeking. Violence Women 2011; 17(8): 1086–1102. 10.1177/1077801211417151 21890530

[44] Fulu E Jewkes R Roselli T , et al. Prevalence of and factors associated with male perpetration of intimate partner violence: findings from the UN Multi-country Cross-sectional Study on Men and Violence in Asia and the Pacific. Lancet Glob Health 2013; 1(4): e187–207. 10.1016/S2214-109X(13)70074-3 25104345

[45] Murray LK Kane JC Glass N , et al. Effectiveness of the common elements treatment approach (CETA) in reducing intimate partner violence and hazardous alcohol use in Zambia (VATU): a randomized controlled trial. PLOS Med 2020; 17(4): e1003056. 10.1371/journal.pmed.1003056 PMC7164585 32302308

[46] Khanal DR Pandey PR Sharma B. Nepal Human Development Report 2020. Nepal: Government of Nepal and United Nations Development Programme, 2020.

[47] Kanougiya S Sivakami M Daruwalla N , et al. Prevalence, pattern, and predictors of formal help-seeking for intimate partner violence against women: findings from India’s cross-sectional National Family Health Surveys-3 (2005–2006) and 4 (2015–2016). BMC Public Health 2022; 22(1): 2386. 36536339 10.1186/s12889-022-14650-3 PMC9764516

[48] Shawon MSR Hossain FB Ahmed R , et al. Role of women empowerment on mental health problems and care-seeking behavior among married women in Nepal: secondary analysis of nationally representative data. Arch Womens Ment Health 2024; 27(4): 527–536. 38315185 10.1007/s00737-024-01433-5 PMC11230993

[49] Shrestha R Sapkota D Sarraf RR , et al. Perceptions on violence against women and its impacts on mental health and response mechanisms among community-based stakeholders: a qualitative study from Nepal. BMC Womens Health 2024; 24(1): 258. 38658963 10.1186/s12905-024-03064-5 PMC11040903

[50] Silva T Agampodi T Evans M , et al. Barriers to help-seeking from healthcare professionals amongst women who experience domestic violence—a qualitative study in Sri Lanka. BMC Public Health 2022; 22(1): 721. 35410170 10.1186/s12889-022-13116-w PMC9004164

[51] Alsina E Browne JL Gielkens D , et al. Interventions to prevent intimate partner violence: a systematic review and meta-analysis. Violence Women 2024; 30(3–4): 953–980. 10.1177/10778012231183660 PMC10845820 37475456

[52] Ragavan M Iyengar K Wurtz R. Perceptions of options available for victims of physical intimate partner violence in Northern India. Violence Women 2015; 21(5): 652–675. 10.1177/1077801215573332 25780061

[53] Jayatilleke AC Poudel KC Yasuoka J , et al. Intimate partner violence in Sri Lanka. Biosci Trends 2010; 4(3): 90–95. 20592458

[54] Ogbe E Harmon S Van Den Bergh R , et al. A systematic review of intimate partner violence interventions focused on improving social support and/ mental health outcomes of survivors. PLoS One 2020; 15(6): e0235177. 10.1371/journal.pone.0235177 PMC7316294 32584910

[55] Abramsky T Devries KM Michau L , et al. The impact of SASA!, a community mobilisation intervention, on women’s experiences of intimate partner violence: secondary findings from a cluster randomised trial in Kampala, Uganda. J Epidemiol Community Health 2016; 70(8): 818–825. 26873948 10.1136/jech-2015-206665 PMC4975800

