# Supplementary material for: Burden of intimate partner violence, mental health issues, and help-seeking behaviors among women in Nepal
Source: Womens Health (Lond). 2025 Mar 18;21:17455057251326416. doi: 10.1177/17455057251326416 (PMC11921001; doi:10.1177/17455057251326416)
Supplement: sj-docx-2-whe-10.1177_17455057251326416 – Supplemental material for Burden of intimate partner violence, mental health issues, and help-seeking behaviors among women in Nepal [file sj-docx-2-whe-10.1177_17455057251326416.docx]

# **Appendix A**

| **Table A1.** The items used to measure the various forms of intimate partner violence. | |
| --- | --- |
| **Form of violence** | **Items (Questions posed to women regarding whether their current or former husband/intimate partner committed the following):** |
| **Physical violence** | a) pushing, shaking, or throwing something at her  b) slapping her  c) twisting her arm or pulling her hair  d) punching her with his fist or with something that could hurt her  e) kicking, dragging, or beating her up  f) trying to choke or burn her on purpose  g) attacking her with a knife, gun, or other weapon |
| **Sexual violence** | a) physically forcing to have sexual intercourse with him  b) physically forcing to perform any other sexual acts  c) forcing with threats or in any other way to perform sexual acts she did not want to |
| **Emotional violence** | a) humiliating her  b) threatening her  c) insulting or making her feel bad about herself |
| **Controlling behavior** | a) husband/partner jealous if respondent talks with other men  b) accuses respondent of unfaithfulness  c) does not permit to meet female friends  d) insists on knowing where respondent is  e) does not trust respondent with money |

| **Table A2.** Unweighted numbers for sociodemographic and behavioral characteristics of women aged 15-49 (n=5,178). | | |
| --- | --- | --- |
| **Variable** | **Mean (range)** | **SD** |
| **Age (15-49)** | 30.6 (15-49) | 9.3 |
|  | **N** | **%** |
| **Educational level** |  |  |
| No education | 1,458 | 28.2 |
| Primary | 1,721 | 33.2 |
| Secondary | 1,855 | 35.8 |
| Higher than secondary | 144 | 2.8 |
| **Employment status** |  |  |
| Currently employed | 3,414 | 65.9 |
| Worked in the past 12 months (but not currently) | 641 | 12.4 |
| Not employed in the past 12 months | 1,123 | 21.7 |
| **Province of residence** |  |  |
| Koshi | 804 | 15.5 |
| Madhesh | 819 | 15.8 |
| Bagmati | 767 | 14.8 |
| Gandaki | 610 | 11.8 |
| Lumbini | 777 | 15.0 |
| Karnali | 701 | 13.6 |
| Sudurpashchim | 700 | 13.5 |
| **Type of place of residence** |  |  |
| Urban | 2,745 | 53.0 |
| Rural | 2,433 | 47.0 |
| **Ethnicity** |  |  |
| Brahmin/Chhetri (Hill/Terai) | 1,744 | 33.7 |
| Other terai caste | 562 | 10.8 |
| Dalit (Hill/Terai) | 859 | 16.7 |
| Janajati/Newar (Hill/Terai) | 1,854 | 35.7 |
| Muslim | 154 | 3.0 |
| Other | 5 | 0.1 |
| **Marital status** |  |  |
| Never in union | 801 | 15.5 |
| Married or living with partner | 4,211 | 81.3 |
| **Marital status** |  |  |
| Widowed | 106 | 2.0 |
| Divorced or separated | 60 | 1.2 |
| **Table A2** (continued). Unweighted numbers for sociodemographic and behavioral characteristics of women aged 15-49 (n=5,178). | | |
| **Variable** | **Mean (range)** | **SD** |
| **Household wealth** ^a^ |  |  |
| Poor | 2,556 | 49.4 |
| Middle | 1,942 | 37.5 |
| Rich | 680 | 13.1 |
| **Partner drunk alcohol in the last month** |  |  |
| No | 2,181 | 48.2 |
| Yes | 2,342 | 51.8 |
| **Frequency of partner being drunk in the last month** |  |  |
| Never | 719 | 30.7 |
| Sometimes | 1,302 | 55.6 |
| Often | 321 | 13.7 |
| **Women consumed alcohol in the last month** |  |  |
| Not consumed | 4,612 | 89.1 |
| 1-10 days in the past month | 422 | 8.1 |
| 11-24 days in the past month | 55 | 1.1 |
| 25 days to daily in the past month | 89 | 1.7 |
| ^a^ Population is divided into five equal parts based on wealth, each comprising approximately 20% of the population. In this table, the first two wealth quintiles are combined into the “poor” category, the third and fourth wealth quintile are grouped together as “middle”, and the fifth wealth quintile is classified as “rich”. | | |

| **Table A3.** Unweighted numbers for experience of mental health problems and help-seeking for IPV among women aged 15-49 (n=5,178). | | |
| --- | --- | --- |
| **Variable** | **N** | **%** |
| **Mental health problems** |  |  |
| Anxiety symptoms over the last 2 weeks (yes) | 1,474 | 28.5 |
| Mild anxiety | 1,073 | 72.8 |
| Moderate anxiety | 318 | 21.6 |
| Severe anxiety | 83 | 5.6 |
| Depressive symptoms over the last 2 weeks (yes) | 1,155 | 22.3 |
| Mild depression | 834 | 72.2 |
| Moderate depression | 230 | 19.9 |
| Moderately severe depression | 68 | 5.9 |
| Severe depression | 23 | 2.0 |
| Suicidal ideation over the last 2 weeks (yes) | 398 | 7.7 |
| **Mental health problems among those who have experienced IPV in the past 12 months** |  |  |
| Anxiety symptoms over the last 2 weeks (yes) | 650 | 42.3 |
| Mild anxiety | 450 | 69.2 |
| Moderate anxiety | 154 | 23.7 |
| Severe anxiety | 46 | 7.1 |
| Depressive symptoms over the last 2 weeks (yes) | 522 | 33.9 |
| Mild depression | 351 | 67.2 |
| Moderate depression | 119 | 22.8 |
| Moderately severe depression | 37 | 7.1 |
| Severe depression | 15 | 2.9 |
| Suicidal ideation over the last 2 weeks (yes) | 227 | 14.7 |
| **Help-seeking for IPV** ^a^ |  |  |
| No | 910 | 72.9 |
| Yes | 339 | 27.1 |
| Informal help ^b^ | 308 | 90.9 |
| Formal help ^c^ | 10 | 2.9 |
| Both (informal & formal help) | 21 | 6.2 |
| ^a^ Out of those who had experienced physical or sexual IPV, and were asked about help-seeking (n=1,249)  ^b^ natal family, husband’s or partner’s family, partner, friends, neighbors, religious leaders, or others  ^c^ doctors, police, lawyers, or social work organizations | | |

| **Table A4.1.** The association between mental health problems and predictors, including sociodemographic and behavioral factors, and experience of IPV in the past 12 months among Nepalese women. | | | | | | | | | | | | |
| --- | --- | --- | --- | --- | --- | --- | --- | --- | --- | --- | --- | --- |
|  | **Anxiety (yes)** | | | | **Depression (yes)** | | | | **Suicidal ideation (yes)** | | | |
|  | **Unadjusted OR (95% CI)** | **p-value** | **aOR**^a^ **(95% CI)** | **p-value** | **Unadjusted OR (95% CI)** | **p-value** | **aOR**^b^ **(95% CI)** | **p-value** | **Unadjusted OR (95% CI)** | **p-value** | **aOR**^c^ **(95% CI)** | **p-**  **value** |
| **Age** |  |  |  |  |  |  |  |  |  |  |  |  |
| <24 | *reference* | | | | *reference* | | | | *reference* | | | |
| 24 and over | 1.16 (0.98-1.37) | 0.094 | 0.97 (0.70-1.35) | 0.873 | 1.10 (0.91-1.33) | 0.328 | - | - | 1.08 (0.79-1.47) | 0.645 | - | - |
| **Educational level** |  |  |  |  |  |  |  |  |  |  |  |  |
| No education | *reference* | | | | *reference* | | | | *reference* | | | |
| Primary | 0.84 (0.68-1.04) | 0.112 | 0.85 (0.63-1.15) | 0.289 | 0.85 (0.68-1.05) | 0.131 | 0.96 (0.70-1.32) | 0.811 | 0.97 (0.72-1.31) | 0.843 | 1.05 (0.67-1.65) | 0.823 |
| Secondary or higher ^d^ | 0.77 (0.62-0.95) | 0.016 | 0.76 (0.51-1.13) | 0.174 | 0.74 (0.59-0.94) | 0.012 | 0.82 (0.54-1.23) | 0.327 | 0.54 (0.39-0.76) | <0.001 | 1.14 (0.62-2.09) | 0.680 |
| **Employment status** |  |  |  |  |  |  |  |  |  |  |  |  |
| Not employed ^e^ | *reference* | | | | *reference* | | | | *reference* | | | |
| Currently employed | 1.14 (1.00-1.29) | 0.050 | 0.91 (0.69-1.20) | 0.496 | 1.06 (0.93-1.22) | 0.380 | - | - | 1.01 (0.81-1.25) | 0.948 | - | - |
| **Province of residence** |  |  |  |  |  |  |  |  |  |  |  |  |
| Bagmati | *reference* | | | | *reference* | | | | *reference* | | | |
| Koshi | 1.34 (0.98-1.84) | 0.066 | 1.84 (1.13-3.00) | 0.015 | 1.69 (1.20-2.36) | 0.002 | 1.99 (1.26-3.15) | 0.003 | 1.78 (1.07-2.95) | 0.025 | 1.60 (0.72-3.58) | 0.251 |
| **Table A4.1** (continued). The association between mental health problems and predictors, including sociodemographic and behavioral factors, and experience of IPV in the past 12 months among Nepalese women. | | | | | | | | | | | | |
|  | **Anxiety (yes)** | | | | **Depression (yes)** | | | | **Suicidal ideation (yes)** | | | |
|  | **Unadjusted OR (95% CI)** | **p-value** | **aOR**^a^ **(95% CI)** | **p-value** | **Unadjusted OR (95% CI)** | **p-value** | **aOR**^b^ **(95% CI)** | **p-value** | **Unadjusted OR (95% CI)** | **p-value** | **aOR**^c^ **(95% CI)** | **p-**  **value** |
| **Province of residence** |  |  |  |  |  |  |  |  |  |  |  |  |
| Madhesh | 1.04 (0.73-1.50) | 0.812 | 0.84 (0.45-1.55) | 0.576 | 1.09 (0.73-1.63) | 0.675 | 0.89 (0.49-1.61) | 0.689 | 1.33 (0.75-2.35) | 0.326 | 1.09 (0.47-2.53) | 0.847 |
| Gandaki | 0.86 (0.60-1.23) | 0.402 | 1.19 (0.78-1.83) | 0.422 | 0.98 (0.66-1.45) | 0.917 | 0.95 (0.59-1.52) | 0.829 | 1.11 (0.59-2.08) | 0.757 | 1.03 (0.50-2.12) | 0.933 |
| Lumbini | 1.06 (0.76-1.49) | 0.723 | 1.24 (0.77-2.00) | 0.378 | 1.06 (0.74-1.53) | 0.753 | 0.97 (0.61-1.55) | 0.895 | 1.41 (0.82-2.42) | 0.214 | 1.31 (0.62-2.75) | 0.481 |
| Karnali | 1.66 (1.19-2.31) | 0.003 | 2.31 (1.39-3.83) | 0.001 | 1.94 (1.41-2.67) | <0.001 | 2.38 (1.48-3.84) | <0.001 | 2.62 (1.61-4.24) | <0.001 | 2.11 (1.00-4.42) | 0.049 |
| Sudurpaschim | 1.38 (0.98-1.94) | 0.065 | 1.88 (1.12-3.17) | 0.017 | 1.27 (0.90-1.79) | 0.173 | 1.37 (0.84-2.23) | 0.213 | 1.61 (0.96-2.70) | 0.071 | 1.57 (0.68-3.63) | 0.286 |
| **Type of place of residence** |  |  |  |  |  |  |  |  |  |  |  |  |
| Urban | *reference* | | | | *reference* | | | | *reference* | | | |
| Rural | 1.02 (0.85-1.22) | 0.872 | - | - | 1.17 (0.96-1.44) | 0.124 | 1.11 (0.85-1.45) | 0.451 | 1.27 (0.96-1.69) | 0.094 | 1.00 (0.68-1.46) | 0.996 |
| **Ethnicity** |  |  |  |  |  |  |  |  |  |  |  |  |
| Brahmin/  Chhetri  (hill & terai) | *reference* | | | | *reference* | | | | *reference* | | | |
| Janajati & Newar | 0.90 (0.74-1.10) | 0.307 | 0.86 (0.62-1.20) | 0.380 | 0.91 (0.74-1.13) | 0.387 | 0.91 (0.65-1.28) | 0.596 | 0.80 (0.59-1.10) | 0.178 | 0.64 (0.39-1.07) | 0.088 |
| Dalit & Muslim ^f^ | 1.29 (1.00-1.67) | 0.050 | 1.01 (0.65-1.55) | 0.975 | 1.30 (1.01-1.68) | 0.045 | 1.18 (0.77-1.80) | 0.449 | 1.74 (1.22-2.45) | 0.002 | 1.44 (0.80-2.60) | 0.223 |
| **Table A4.1** (continued). The association between mental health problems and predictors, including sociodemographic and behavioral factors, and experience of IPV in the past 12 months among Nepalese women. | | | | | | | | | | | | |
|  | **Anxiety (yes)** | | | | **Depression (yes)** | | | | **Suicidal ideation (yes)** | | | |
|  | **Unadjusted OR (95% CI)** | **p-value** | **aOR**^a^ **(95% CI)** | **p-value** | **Unadjusted OR (95% CI)** | **p-value** | **aOR**^b^ **(95% CI)** | **p-value** | **Unadjusted OR (95% CI)** | **p-value** | **aOR**^c^ **(95% CI)** | **p-**  **value** |
| **Ethnicity** |  |  |  |  |  |  |  |  |  |  |  |  |
| Other ^g^ | 1.00 (0.75-1.32) | 0.988 | 1.06 (0.59-1.91) | 0.839 | 0.87 (0.63-1.20) | 0.383 | 0.95 (0.51-1.76) | 0.868 | 1.11 (0.69-1.76) | 0.671 | 1.25 (0.55-2.85) | 0.596 |
| **Marital status** |  |  |  |  |  |  |  |  |  |  |  |  |
| Married | 1.25 (1.01-1.54) | 0.044 | 0.41 (0.13-1.36) | 0.147 | 1.32 (1.05-1.66) | 0.018 | 0.35 (0.09-1.33) | 0.123 | 1.67 (1.08-2.57) | 0.021 | 0.70 (0.19-2.64) | 0.598 |
| Widowed/  separated ^h^ | 2.42 (1.57-3.73) | <0.001 | 0.78 (0.22-2.70) | 0.689 | 2.70 (1.76-4.15) | <0.001 | 0.75 (0.18-3.11) | 0.693 | 3.53 (1.78-6.99) | <0.001 | 2.19 (0.49-9.79) | 0.304 |
| **Household wealth** |  |  |  |  |  |  |  |  |  |  |  |  |
| Poor  (0-40%) | *reference* | | | | *reference* | | | | *reference* | | | |
| Middle  (40-80%) | 0.94 (0.79-1.13) | 0.529 | 1.23 (0.94-1.60) | 0.127 | 0.78 (0.64-0.94) | 0.008 | 1.13 (0.85-1.50) | 0.385 | 0.71 (0.53-0.93) | 0.015 | 0.89 (0.62-1.28) | 0.534 |
| Rich  (80-100%) | 0.72 (0.55-0.93) | 0.013 | 1.00 (0.62-1.62) | 0.998 | 0.67 (0.51) | 0.003 | 1.32 (0.78-2.23) | 0.302 | 0.50 (0.31-0.80) | 0.004 | 0.73 (0.31-1.71) | 0.469 |
| **Frequency of partner being drunk** |  |  |  |  |  |  |  |  |  |  |  |  |
| Never | *reference* | | | | *reference* | | | | *reference* | | | |
| Sometimes | 1.55 (1.21-1.98) | <0.001 | 1.27 (0.97-1.65) | 0.077 | 1.42 (1.09-1.86) | 0.010 | 1.20 (0.90-1.61) | 0.211 | 1.73 (1.10-2.74) | 0.019 | 1.19 (0.75-1.90) | 0.455 |
| Often | 4.14 (2.89-5.94) | <0.001 | 2.18 (1.46-3.25) | <0.001 | 3.60 (2.44-5.30) | <0.001 | 2.03 (1.32-3.14) | 0.001 | 4.96 (3.08-7.98) | <0.001 | 1.96 (1.08-3.57) | 0.027 |
| **Table A4.1** (continued). The association between mental health problems and predictors, including sociodemographic and behavioral factors, and experience of IPV in the past 12 months among Nepalese women. | | | | | | | | | | | | |
|  | **Anxiety (yes)** | | | | **Depression (yes)** | | | | **Suicidal ideation (yes)** | | | |
|  | **Unadjusted OR (95% CI)** | **p-value** | **aOR**^a^ **(95% CI)** | **p-value** | **Unadjusted OR (95% CI)** | **p-value** | **aOR**^b^ **(95% CI)** | **p-value** | **Unadjusted OR (95% CI)** | **p-value** | **aOR**^c^ **(95% CI)** | **p-**  **value** |
| **Woman drinking** |  |  |  |  |  |  |  |  |  |  |  |  |
| Not consumed | *reference* | | | | *reference* | | | | *reference* | | | |
| 1-10 days | 1.34 (1.02-1.76) | 0.038 | 0.92 (0.64-1.32) | 0.644 | 1.09 (0.81-1.46) | 0.554 | - | - | 1.25 (0.76-2.07) | 0.384 | - | - |
| 11 days to daily | 1.25 (0.81-1.93) | 0.320 | 1.05 (0.55-1.97) | 0.892 | 0.81 (0.51-1.29) | 0.375 | - | - | 1.09 (0.59-2.04) | 0.779 | - | - |
| **Experienced any type of IPV in the last 12 months (yes)** | 2.49 (2.10-2.96) | <0.001 | 1.52 (0.90-2.55) | 0.118 | 2.51 (2.09-3.01) | <0.001 | 0.75 (0.45-1.25) | 0.277 | 3.75 (2.81-5.00) | <0.001 | 0.95 (0.48-1.88) | 0.890 |
| Physical IPV (Yes) | 2.76 (2.20-3.46) | <0.001 | 1.06 (0.71-1.57) | 0.790 | 2.94 (2.30-3.76) | <0.001 | 1.07 (0.72-1.60) | 0.725 | 4.52 (3.25-6.28) | <0.001 | 1.55 (0.92-2.61) | 0.100 |
| Sexual IPV (Yes) | 6.06 (4.21-8.71) | <0.001 | 2.88 (1.67-4.95) | <0.001 | 4.70 (3.39-6.52) | <0.001 | 2.12 (1.32-3.41) | 0.002 | 5.56 (3.62-8.53) | <0.001 | 1.63 (0.90-2.96) | 0.108 |
| Emotional IPV (Yes) | 4.97 (3.86-6.40) | <0.001 | 3.00 (2.08-4.31) | <0.001 | 4.83 (3.71-6.29) | <0.001 | 3.09 (2.19-4.37) | <0.001 | 5.16 (3.88-6.86) | <0.001 | 1.91 (1.20-3.04) | 0.006 |
| Controlling behavior (Yes) | 2.28 (1.90-2.74) | <0.001 | 1.03 (0.65-1.62) | 0.896 | 2.42 (2.02-2.89) | <0.001 | 1.88 (1.21-2.92) | 0.005 | 3.44 (2.56-4.63) | <0.001 | 2.24 (1.25-4.02) | 0.007 |
| Abbreviations: OR – odds ratio; CI – confidence interval; aOR – adjusted odds ratio  ^a^ Adjusted for: age, education, employment, province, marital status, frequency of partner being drunk, and woman drinking  ^b^ Adjusted for education, province, type of residence (urban/rural), ethnicity, marital status, household wealth, and frequency of partner being drunk  ^c^ Adjusted for education, type of residence (urban/rural), marital status, household wealth, and frequency of partner being drunk  ^d^ Secondary and higher than secondary education merged to increase statistical power  ^e^ Including those who have worked in the past 12 months, but are not currently employed, and those who have not been employed in the past 12 month  ^f^ Dalit and Muslim merged as one group to increase statistical power  ^g^ Other terai caste and other non-specified ethnicities  ^h^ Widowed, divorced, and separated merged as one group to increase statistical power | | | | | | | | | | | | |

| **Table A4.2.** The association between mental health problems and predictors, including sociodemographic and behavioral factors, and ever experienced IPV among Nepalese women. | | | | | | | | | | | | |
| --- | --- | --- | --- | --- | --- | --- | --- | --- | --- | --- | --- | --- |
|  | **Anxiety (yes)** | | | | **Depression (yes)** | | | | **Suicidal ideation (yes)** | | | |
|  | **Unadjusted OR (95% CI)** | **p-value** | **aOR**^a^ **(95% CI)** | **p-value** | **Unadjusted OR (95% CI)** | **p-value** | **aOR**^b^ **(95% CI)** | **p-value** | **Unadjusted OR (95% CI)** | **p-value** | **aOR**^c^ **(95% CI)** | **p-**  **value** |
| **Age** |  |  |  |  |  |  |  |  |  |  |  |  |
| <24 | *reference* | | | | *reference* | | | | *reference* | | | |
| 24 and over | 1.16 (0.98-1.37) | 0.094 | 0.88 (0.64-1.22) | 0.443 | 1.10 (0.91-1.33) | 0.328 | - | - | 1.08 (0.79-1.47) | 0.645 | - | - |
| **Educational level** |  |  |  |  |  |  |  |  |  |  |  |  |
| No education | *reference* | | | | *reference* | | | | *reference* | | | |
| Primary | 0.84 (0.68-1.04) | 0.112 | 0.86 (0.64-1.16) | 0.322 | 0.85 (0.68-1.05) | 0.131 | 1.00 (0.73-1.38) | 0.980 | 0.97 (0.72-1.31) | 0.843 | 1.07 (0.68-1.68) | 0.772 |
| Secondary or higher ^d^ | 0.77 (0.62-0.95) | 0.016 | 0.76 (0.51-1.15) | 0.191 | 0.74 (0.59-0.94) | 0.012 | 0.84 (0.55-1.28) | 0.424 | 0.54 (0.39-0.76) | <0.001 | 1.16 (0.62-2.18) | 0.636 |
| **Employment status** |  |  |  |  |  |  |  |  |  |  |  |  |
| Not employed ^e^ | *reference* | | | | *reference* | | | | *reference* | | | |
| Currently employed | 1.14 (1.00-1.29) | 0.050 | 0.88 (0.67-1.17) | 0.374 | 1.06 (0.93-1.22) | 0.380 | - | - | 1.01 (0.81-1.25) | 0.948 | - | - |
| **Province of residence** |  |  |  |  |  |  |  |  |  |  |  |  |
| Bagmati | *reference* | | | | *reference* | | | | *reference* | | | |
| Koshi | 1.34 (0.98-1.84) | 0.066 | 1.78 (1.10-2.87) | 0.018 | 1.69 (1.20-2.36) | 0.002 | 1.95 (1.24-3.09) | 0.004 | 1.78 (1.07-2.95) | 0.025 | 1.53 (0.71-3.33) | 0.279 |
| **Table A4.2** (continued). The association between mental health problems and predictors, including sociodemographic and behavioral factors, and ever experienced IPV among Nepalese women. | | | | | | | | | | | | |
|  | **Anxiety (yes)** | | | | **Depression (yes)** | | | | **Suicidal ideation (yes)** | | | |
|  | **Unadjusted OR (95% CI)** | **p-value** | **aOR**^a^ **(95% CI)** | **p-value** | **Unadjusted OR (95% CI)** | **p-value** | **aOR**^b^ **(95% CI)** | **p-value** | **Unadjusted OR (95% CI)** | **p-value** | **aOR**^c^ **(95% CI)** | **p-**  **value** |
| **Province of residence** |  |  |  |  |  |  |  |  |  |  |  |  |
| Madhesh | 1.04 (0.73-1.50) | 0.812 | 0.83 (0.46-1.50) | 0.534 | 1.09 (0.73-1.63) | 0.675 | 0.87 (0.48-1.56) | 0.631 | 1.33 (0.75-2.35) | 0.326 | 1.01 (0.44-2.32) | 0.977 |
| Gandaki | 0.86 (0.60-1.23) | 0.402 | 1.10 (0.71-1.72) | 0.664 | 0.98 (0.66-1.45) | 0.917 | 0.90 (0.54-1.49) | 0.676 | 1.11 (0.59-2.08) | 0.757 | 0.99 (0.47-2.09) | 0.978 |
| Lumbini | 1.06 (0.76-1.49) | 0.723 | 1.19 (0.74-1.89) | 0.473 | 1.06 (0.74-1.53) | 0.753 | 0.94 (0.58-1.50) | 0.787 | 1.41 (0.82-2.42) | 0.214 | 1.28 (0.61-2.68) | 0.505 |
| Karnali | 1.66 (1.19-2.31) | 0.003 | 2.18 (1.32-3.59) | 0.002 | 1.94 (1.41-2.67) | <0.001 | 2.39 (1.49-3.83) | <0.001 | 2.62 (1.61-4.24) | <0.001 | 2.17 (1.05-4.50) | 0.037 |
| Sudurpaschim | 1.38 (0.98-1.94) | 0.065 | 1.85 (1.10-3.12) | 0.021 | 1.27 (0.90-1.79) | 0.173 | 1.39 (0.85-2.28) | 0.191 | 1.61 (0.96-2.70) | 0.071 | 1.73 (0.75-3.96) | 0.196 |
| **Type of place of residence** |  |  |  |  |  |  |  |  |  |  |  |  |
| Urban | *reference* | | | | *reference* | | | | *reference* | | | |
| Rural | 1.02 (0.85-1.22) | 0.872 | - | - | 1.17 (0.96-1.44) | 0.124 | 1.13 (0.87-1.46) | 0.367 | 1.27 (0.96-1.69) | 0.094 | 0.98 (0.68-1.43) | 0.935 |
| **Ethnicity** |  |  |  |  |  |  |  |  |  |  |  |  |
| Brahmin/Chhetri (hill & terai) | *reference* | | | | *reference* | | | | *reference* | | | |
| Janajati & Newar | 0.90 (0.74-1.10) | 0.307 | 0.84 (0 .60-1.18) | 0.322 | 0.91 (0.74-1.13) | 0.387 | 0.92 (0.65-1.29) | 0.623 | 0.80 (0.59-1.10) | 0.178 | 0.68 (0.41-1.14) | 0.140 |
| Dalit & Muslim ^f^ | 1.29 (1.00-1.67) | 0.050 | 1.00 (0.65-1.54) | 0.988 | 1.30 (1.01-1.68) | 0.045 | 1.17 (0.76-1.80) | 0.474 | 1.74 (1.22-2.45) | 0.002 | 1.49 (0.82-2.70) | 0.189 |
| **Table A4.2** (continued). The association between mental health problems and predictors, including sociodemographic and behavioral factors, and ever experienced IPV among Nepalese women. | | | | | | | | | | | | |
|  | **Anxiety (yes)** | | | | **Depression (yes)** | | | | **Suicidal ideation (yes)** | | | |
|  | **Unadjusted OR (95% CI)** | **p-value** | **aOR**^a^ **(95% CI)** | **p-value** | **Unadjusted OR (95% CI)** | **p-value** | **aOR**^b^ **(95% CI)** | **p-value** | **Unadjusted OR (95% CI)** | **p-value** | **aOR**^c^ **(95% CI)** | **p-**  **value** |
| **Ethnicity** |  |  |  |  |  |  |  |  |  |  |  |  |
| Other ^g^ | 1.00 (0.75-1.32) | 0.988 | 0.99 (0.56-1.72) | 0.959 | 0.87 (0.63-1.20) | 0.383 | 0.91 (0.50-1.64) | 0.744 | 1.11 (0.69-1.76) | 0.671 | 1.16 (0.52-2.59) | 0.711 |
| **Marital status** |  |  |  |  |  |  |  |  |  |  |  |  |
| Never in union | *reference* | | | | *reference* | | | | *reference* | | | |
| Married | 1.25 (1.01-1.54) | 0.044 | 0.43 (0.13-1.41) | 0.163 | 1.32 (1.05-1.66) | 0.018 | 0.33 (0.10-1.12) | 0.074 | 1.67 (1.08-2.57) | 0.021 | 0.89 (0.24-3.22) | 0.855 |
| Widowed/  separated ^h^ | 2.42 (1.57-3.73) | <0.001 | 0.42 (0.11-1.53) | 0.188 | 2.70 (1.76-4.15) | <0.001 | 0.40 (0.10-1.55) | 0.185 | 3.53 (1.78-6.99) | <0.001 | 1.17 (0.28-5.01) | 0.828 |
| **Household wealth** |  |  |  |  |  |  |  |  |  |  |  |  |
| Poor (0-40%) | *reference* | | | | *reference* | | | | *reference* | | | |
| Middle (40-80%) | 0.94 (0.79-1.13) | 0.529 | 1.23 (0.94-1.61) | 0.133 | 0.78 (0.64-0.94) | 0.008 | 1.13 (0.85-1.50) | 0.389 | 0.71 (0.53-0.93) | 0.015 | 0.90 (0.63-1.28) | 0.563 |
| Rich (80-100%) | 0.72 (0.55-0.93) | 0.013 | 1.00 (0.65-1.54) | 0.989 | 0.67 (0.51) | 0.003 | 1.36 (0.81-2.27) | 0.240 | 0.50 (0.31-0.80) | 0.004 | 0.75 (0.33-1.73) | 0.504 |
| **Frequency of partner being drunk** |  |  |  |  |  |  |  |  |  |  |  |  |
| Never | *reference* | | | | *reference* | | | | *reference* | | | |
| Sometimes | 1.55 (1.21-1.98) | <0.001 | 1.24 (0.95-1.61) | 0.113 | 1.42 (1.09-1.86) | 0.010 | 1.56 (0.86-1.54) | 0.330 | 1.73 (1.10-2.74) | 0.019 | 1.09 (0.68-1.75) | 0.728 |

| **Table A4.2** (continued). The association between mental health problems and predictors, including sociodemographic and behavioral factors, and ever experienced IPV among Nepalese women. | | | | | | | | | | | | |
| --- | --- | --- | --- | --- | --- | --- | --- | --- | --- | --- | --- | --- |
|  | **Anxiety (yes)** | | | | **Depression (yes)** | | | | **Suicidal ideation (yes)** | | | |
|  | **Unadjusted OR (95% CI)** | **p-value** | **aOR**^a^ **(95% CI)** | **p-value** | **Unadjusted OR (95% CI)** | **p-value** | **aOR**^b^ **(95% CI)** | **p-value** | **Unadjusted OR (95% CI)** | **p-value** | **aOR**^c^ **(95% CI)** | **p-**  **value** |
| **Frequency of partner being drunk** |  |  |  |  |  |  |  |  |  |  |  |  |
| Often | 4.14 (2.89-5.94) | <0.001 | 2.05 (1.36-3.09) | 0.001 | 3.60 (2.44-5.30) | <0.001 | 1.81 (1.16-2.80) | 0.008 | 4.96 (3.08-7.98) | <0.001 | 1.50 (0.81-2.75) | 0.194 |
| **Woman drinking** |  |  |  |  |  |  |  |  |  |  |  |  |
| Not consumed | *reference* | | | | *reference* | | | | *reference* | | | |
| 1-10 days | 1.34 (1.02-1.76) | 0.038 | 0.95 (0.65-1.37) | 0.765 | 1.09 (0.81-1.46) | 0.554 | - | - | 1.25 (0.76-2.07) | 0.384 | - | - |
| 11 days to daily | 1.25 (0.81-1.93) | 0.320 | 1.04 (0.56-1.93) | 0.893 | 0.81 (0.51-1.29) | 0.375 | - | - | 1.09 (0.59-2.04) | 0.779 | - | - |
| **Ever experienced any type of IPV (yes)** | 2.73 (2.33-3.20) | <0.001 | 1.20 (0.78- 1.86) | 0.412 | 2.85 (2.39-3.39) | <0.001 | 0.89 (0.54- 1.45) | 0.634 | 4.57 (3.45-6.05) | <0.001 | 1.24 (0.61-2.52) | 0.553 |
| Physical IPV (Yes) | 2.66 (2.24-3.16) | <0.001 | 1.01 (0.69- 1.47) | 0.971 | 2.75 (2.26-3.35) | <0.001 | 1.18 (0.79-1.76) | 0.424 | 4.14 (3.15-5.43) | <0.001 | 1.27 (0.72-2.23) | 0.410 |
| Sexual IPV (Yes) | 4.70 (3.60-6.14) | <0.001 | 2.21 (1.44- 3.39) | <0.001 | 4.03 (3.17-5.13) | <0.001 | 1.61 (1.09-2.37) | 0.017 | 5.57 (4.12-7.53) | <0.001 | 1.68 (1.08-2.61) | 0.021 |
| Emotional IPV (Yes) | 4.18 (3.36-5.20) | <0.001 | 2.22 (1.58-3.12) | <0.001 | 4.51 (3.50-5.80) | <0.001 | 2.65 (1.82- 3.87) | <0.001 | 5.73 (4.30-7.64) | <0.001 | 2.30 (1.42-3.74) | 0.001 |
| Controlling behavior (Yes) | 2.36 (1.98-2.82) | <0.001 | 1.31 (0.93-1.86) | 0.123 | 2.58 (2.17-3.06) | <0.001 | 1.56 (1.05-2.32) | 0.028 | 3.91 (2.89-5.29) | <0.001 | 1.94 (1.04-3.60) | 0.036 |

| **Table A4.2** (continued). The association between mental health problems and predictors, including sociodemographic and behavioral factors, and ever experienced IPV among Nepalese women. |
| --- |

| Abbreviations: OR – odds ratio; CI – confidence interval; aOR – adjusted odds ratio  ^a^ Adjusted for: age, education, employment, province, marital status, frequency of partner being drunk, and woman drinking  ^b^ Adjusted for education, province, type of residence (urban/rural), ethnicity, marital status, household wealth, and frequency of partner being drunk  ^c^ Adjusted for education, type of residence (urban/rural), marital status, household wealth, and frequency of partner being drunk  ^d^ Secondary and higher than secondary education merged to increase statistical power  ^e^ Including those who have worked in the past 12 months, but are not currently employed, and those who have not been employed in the past 12 month  ^f^ Dalit and Muslim merged as one group to increase statistical power  ^g^ Other terai caste and other non-specified ethnicities  ^h^ Widowed, divorced, and separated merged as one group to increase statistical power |
| --- |

| **Table A5.** The association between sociodemographic and behavioral factor and seeking help to stop the violence among women who have experienced physical or sexual IPV (n=1,249). | | | | |
| --- | --- | --- | --- | --- |
|  | **Have sought help for IPV (yes)** | | | |
|  | **Unadjusted OR**  **(95% CI)** | **p-value** | **aOR** ^a^  **(95% CI)** | **p-value** |
| **Employment status** |  |  |  |  |
| Not employed | *reference* | | *reference* | |
| Currently employed | 1.46 (1.10-1.94) | 0.008 | 1.49 (0.96-2.33) | 0.076 |
| **Province of residence** |  |  |  |  |
| Bagmati | *reference* | | *reference* | |
| Koshi | 0.96 (0.49) | 0.915 | 1.22 (0.59-2.55) | 0.593 |
| Madhesh | 0.70 (0.36-1.36) | 0.290 | 1.01 (0.44-2.34) | 0.975 |
| Gandaki | 0.77 (0.38-1.55) | 0.458 | 0.78 (0.34-1.79) | 0.559 |
| Lumbini | 0.83 (0.43-1.59) | 0.571 | 1.05 (0.50-2.20) | 0.902 |
| Karnali | 0.79 (0.39-1.60) | 0.507 | 0.56 (0.24-1.32) | 0.185 |
| Sudurpaschim | 0.40 (0.19-0.84) | 0.015 | 0.39 (0.17-0.89) | 0.025 |
| **Ethnicity** |  |  |  |  |
| Brahmin/Chhetri (hill & terai) | *reference* | | *reference* | |
| Janajati & Newar | 0.89 (0.59-1.34) | 0.583 | 0.88 (0.51-1.54) | 0.663 |
| Dalit & Muslim | 0.54 (0.33-0.89) | 0.011 | 0.62 (0.33-1.15) | 0.128 |
| Other ^b^ | 0.68 (-0.40-1.17) | 0.165 | 0.69 (0.32-1.45) | 0.324 |
| **Marital status** |  |  |  |  |
| Never in union | *reference* | | *reference* | |
| Married | 0.94 (0.49-1.81) | 0.855 | 0.48 (0.11-2.13) | 0.334 |
| Widowed/separated | 2.40 (0.92-6.26) | 0.075 | 0.71 (0.14-3.58) | 0.685 |
| **Frequency of partner being drunk** |  |  |  |  |
| Never | *reference* | | *reference* | |
| Sometimes | 1.14 (0.63-2.05) | 0.659 | 1.01 (0.57-1.77) | 0.973 |
| Often | 2.28 (1.21-4.29) | 0.011 | 2.17 (1.17-4.02) | 0.014 |
| **Woman drinking** |  |  |  |  |
| Not consumed | *reference* | | *reference* | |
| 1-10 days | 1.17 (0.72-1.90) | 0.532 | 0.82 (0.43-1.58) | 0.548 |
| 11 days to daily | 1.95 (0.95-4.00) | 0.070 | 1.50 (0.57-3.98) | 0.410 |
| ^a^ Adjusted for: employment, province, ethnicity, marital status, frequency of partner being drunk, and frequency of woman drinking  ^b^ Other terai caste and other non-specified ethnicities | | | | |

| **Table A6.** Factors associated with help-seeking for mental health problems among Nepalese women who have experienced IPV in the past 12 months (n=1,541). | | | | |
| --- | --- | --- | --- | --- |
|  | **Have sought help for mental health problems (yes)** | | | |
|  | **Unadjusted OR**  **(95% CI)** | **p-value** | **aOR** a  **(95% CI)** | **p-value** |
| **Age** |  |  |  |  |
| <24 | *reference* | | *reference* | |
| 24 or older | 0.95 (0.76-1.20) | 0.660 | **-** | **-** |
| **Educational level** |  |  |  |  |
| No education | *reference* | | *reference* | |
| Primary | 0.85 (0.65-1.11) | 0.233 | **-** | **-** |
| Secondary or higher | 1.03 (0.80-1.34) | 0.800 | **-** | **-** |
| **Employment status** |  |  |  |  |
| Not employed | *reference* | | *reference* | |
| Currently employed | 1.02 (0.86-1.20) | 0.854 | **-** | **-** |
| **Province of residence** |  |  |  |  |
| Bagmati | *reference* | | *reference* | |
| Koshi | 1.52 (0.97-2.38) | 0.066 | 1.64 (0.93-2.87) | 0.084 |
| Madhesh | 1.33 (0.81-2.19) | 0.254 | 1.71 (0.87- 3.36) | 0.121 |
| Gandaki | 1.08 (0.63-1.86) | 0.783 | 1.46 (0.77- 2.76) | 0.247 |
| Lumbini | 1.27 (0.79-2.05) | 0.318 | 1.75 (0.99-3.10) | 0.055 |
| Karnali | 1.20 (0.79-1.82) | 0.392 | 1.46 (0.82-2.59) | 0.197 |
| Sudurpaschim | 1.43 (0.88-2.32) | 0.144 | 2.09 (1.11-3.96) | 0.023 |
| **Type of place of residence** |  |  |  |  |
| Urban | *reference* | | *reference* | |
| Rural | 0.87 (0.66-1.13) | 0.295 |  |  |
| **Ethnicity** |  |  |  |  |
| Brahmin/Chhetri (hill & terai) | *reference* | | *reference* | |
| Janajati & Newar | 1.10 (0.86-1.41) | 0.429 | 1.31 (0.89- 1.94) | 0.174 |
| Dalit & Muslim | 1.13 (0.84-1.51) | 0.423 | 1.02 (0.66-1.60) | 0.920 |
| Other ^b^ | 1.36 (0.90-2.04) | 0.139 | 1.25 (0.66-2.36) | 0.495 |
| **Marital status** |  |  |  |  |
| Never in union | *reference* | | *reference* | |
| Married | 1.07 (0.81-1.42) | 0.628 | 1.39 (0.52-3.73) | 0.512 |
| Widowed/separated | 1.66 (0.89-3.08) | 0.108 | 2.88 (0.90-9.21) | 0.074 |
| **Household wealth** |  |  |  |  |
| Poor (0-40%) | *reference* | | *reference* | |
| Middle (40-80%) | 1.34 (1.07-1.69) | 0.012 | 1.23 (0.89-1.70) | 0.205 |
| **Table A6** (continued). Factors associated with help-seeking for mental health problems among Nepalese women who have experienced IPV in the past 12 months (n=1,541). | | | | |
|  | **Have sought help for mental health problems (yes)** | | | |
|  | **Unadjusted OR**  **(95% CI)** | **p-value** | **aOR** a  **(95% CI)** | **p-value** |
| **Household wealth** |  |  |  |  |
| Rich (80-100%) | 1.19 (0.89-1.56) | 0.229 | 2.24 (1.39- 3.61) | 0.001 |
| **Frequency of partner being drunk** |  |  |  |  |
| Never | *reference* | | *reference* | |
| Sometimes | 1.06 (0.76-1.48) | 0.748 | 0.77 (0.54-1.10) | 0.157 |
| Often | 1.57 (0.98-2.52) | 0.059 | 0.82 (0.50-1.33) | 0.413 |
| **Woman drinking** |  |  |  |  |
| not consumed | *reference* | | *reference* | |
| 1-10 days | 1.45 (1.01-2.07) | 0.042 | 1.02 (0.67-1.55) | 0.936 |
| 11 days to daily | 0.77 (0.39-1.54) | 0.462 | 1.00 (0.40-2.53) | 0.998 |
| **Anxiety symptoms (yes)** ^c^ | 3.63 (2.90-4.56) | <0.001 | 1.67 (1.14-2.45) | 0.008 |
| **Depressive symptoms (yes)** ^c^ | 3.93 (3.05-5.05) | <0.001 | 1.63 (1.03-2.57) | 0.036 |
| **Suicidal ideation** **(yes)** ^c^ | 3.59 (2.52-5.11) | <0.001 | 1.20 (0.73-1.94) | 0.463 |
| ^a^ Adjusted for: province, ethnicity, marital status, household wealth, frequency of partner being drunk, frequency of woman drinking and anxiety, depressive symptoms, and suicidal ideation among women experiencing IPV in the past 12 months.  ^b^ Other terai caste and other non-specified ethnicities  ^c^ Among women experiencing IPV in the past 12 months (n=1,541) | | | | |
